# Alzheimer's Biomarkers and Visuospatial Cognition in Parkinson's Disease: Modification by α‐Synuclein and Mediation of Age Effects

**DOI:** 10.1002/mdc3.70576

**Published:** 2026-03-06

**Authors:** David Ledingham, Sahana Sathyanarayana, Charlotte B. Stewart, Robyn Iredale, Victoria Foster, Debra Galley, Meher Lad, Mark R. Baker, Nicola Pavese

**Affiliations:** ^1^ Clinical Ageing Research Unit Newcastle University, Campus for Ageing and Vitality Newcastle upon Tyne UK; ^2^ Neurosciences Newcastle Upon Tyne NHS Foundation Trust Newcastle upon Tyne UK; ^3^ Translational and Clinical Research Institute, The Medical School Newcastle University Newcastle upon Tyne UK; ^4^ Department of Nuclear Medicine and PET Centre Aarhus University Hospital Aarhus Denmark

**Keywords:** Alzheimer's disease, biomarkers, Parkinson's disease, pTau181/Aβ42 ratio, seeding amplification assay (SAA), visuospatial cognition

## Abstract

**Background:**

Visuospatial deficits in Parkinson's disease (PD) often precede dementia and complicate daily functioning. Alzheimer's disease (AD) pathology and α‐synuclein aggregation frequently co‐occur in PD, but their combined impact on cognition is unclear.

**Objectives:**

To examine whether AD biomarker burden relates to visuospatial performance in PD, whether this effect differs by α‐synuclein status, and whether AD biomarkers mediate age‐related decline.

**Methods:**

We analyzed 416 participants from the Parkinson's Progression Markers Initiative. AD biomarker burden was indexed by the cerebrospinal fluid pTau181/Aβ42 ratio; α‐synuclein aggregation was assessed using seed amplification assay. Models adjusted for age, sex, education, and motor severity. Sensitivity analyses included genetic stratification and subgroup exclusion.

**Results:**

Higher AD biomarker burden was associated with poorer visuospatial performance and delayed recall. In participants with concurrent biomarker data (*n* = 246), AD burden interacted with α‐synuclein status to predict worse visuospatial outcomes, with the greatest impairment observed in individual's positive for both biomarkers. Mediation analysis indicated that AD biomarker burden accounts for approximately 10–14% of the age effect on visuospatial performance.

**Conclusions:**

AD and α‐synuclein biomarkers show associations consistent with synergistic effects on visuospatial cognition in PD. These findings are exploratory and require replication in pre‐specified independent cohorts. However, if validated, testing both biomarkers could help identify individuals at higher risk of early visuospatial decline and inform hypothesis‐driven stratification in future clinical trials.

Cognitive impairment is a frequent nonmotor manifestation of Parkinson's disease (PD), affecting approximately 25–30% of patients at diagnosis and up to 75% after a decade of disease.^1^, ^2^ Mild cognitive impairment (PDMCI) occurs in a‐bout 30% of de novo cases, and 40–60% of those progress to dementia over time.^3^, ^4^ However, the pattern and rate of decline vary widely, reflecting substantial cognitive heterogeneity across individuals. This variability spans multiple domains, with executive and visuospatial deficits often emerging early, while memory and language changes tend to appear later in the disease course.^5^, ^6^

The biological underpinnings of this heterogeneity are increasingly recognized to include co‐occurring pathologies. In addition to αsynuclein aggregation, many individuals with PD exhibit Alzheimer's disease (AD)–related changes, including a‐myloidβ and tau d‐eposition.^7^, ^8^, ^9^, ^10^, ^11^ Cerebrospinal fluid (CSF) assays now enable in vivo quantification of these processes, with the Elecsys® pTau181/Aβ42 ratio widely adopted as a composite marker of AD‐related pathology.^12^, ^13^ In parallel, α‐synuclein seed amplification assays (SAA) have emerged as highly sensitive and specific markers of Lewy body–type pathology and are now incorporated into disease staging frameworks.^14^, ^15^ Across PD cohorts, lower CSF Aβ42 and elevated tau associate with worse cognition and faster cognitive decline, and autopsy syntheses show frequent AD‐type changes in PD dementia.^7^, ^8^, ^9^, ^10^, ^11^ While SAA has demonstrated excellent diagnostic performance for synucleinopathies, it has not been systematically studied in relation to cognitive outcomes or biomarker interactions.^16^

To date, these two biomarker classes have largely been studied in isolation: Elecsys‐based analyses in PD have not incorporated α‐synuclein SAA^9^ and large‐scale SAA reports have focused on diagnostic performance rather than cognitive outcomes or biomarker synergy.^14^ Supporting evidence from experimental and post‐mortem studies in AD, and from mechanistic and pathological studies in PD, indicates that α‐synuclein can interact with amyloid‐β and tau, promoting mutual aggregation and accelerating neurodegeneration.^17^, ^18^, ^19^, ^20^ Whether similar interactions occur in vivo in PD, and whether they preferentially affect visuospatial cognition, a domain strongly linked to dementia risk, remains unknown.^20^

Previous PD studies have linked AD biomarkers to global and some domain‐specific cognitive decline but visuospatial outcomes have been less systematically examined, and no prior work has tested AD × SAA interactions.^9^, ^21^

We analyzed data from the Parkinson's Progression Markers Initiative (PPMI) to address three questions:

1. Do higher levels of AD biomarker burden relate to worse cognitive performance in PD, with a focus on visuospatial outcomes?
2. Does the effect of AD biomarker burden on cognition differ by α‐synuclein aggregation status (SAA)?
3. Does AD biomarker burden mediate age‐related cognitive decline?

We included mediation analysis because both age and AD pathology are established risk factors for cognitive decline in PD, and we aimed to quantify the extent to which age‐related cognitive changes may operate through AD‐related processes. We prespecified a levodopa‐responsiveness subset (LEDD ≥300 mg/day with ON–OFF UPDRS‐III) to test whether dopaminergic response, could account for variance in cognitive performance independent of AD biomarker burden.

Our design prespecified sporadic PD as the primary inference population and visuospatial outcomes as the primary cognitive domain for interaction testing.

## Methods

### Study Design and Participants

We conducted a cross‐sectional analysis using data from PPMI, a multicenter longitudinal observational study of PD (ClinicalTrials.gov: NCT01141023). PPMI enrolment criteria have been described previously and included individuals with sporadic or genetic PD who were free of dementia at baseline. Briefly, sporadic PD participants were aged ≥30 years, diagnosed within 2 years, and not expected to require dopaminergic therapy within 6 months of enrolment. Genetic PD participants with LRRK2 or GBA variants were eligible if diagnosed within 5 years (≤2 years from 2020 onward), while those with SNCA, PRKN, or PINK1 variants were eligible regardless of diagnosis timing.^22^, ^23^

For this analysis, participants were included if they had a confirmed PD diagnosis and complete data for core clinical variables, including age, sex, education, disease duration, Movement Disorder Society–Unified Parkinson's Disease Rating Scale (MDS‐UPDRS) Parts I–IV, Geriatric Depression Scale (GDS), State–Trait Anxiety Inventory (STAI), and levodopa equivalent daily dose (LEDD), plus cerebrospinal fluid (CSF) biomarkers for Alzheimer's disease (pTau181 and Aβ42) measured on the Roche Elecsys® platform. p‐tau181 was selected because it is the longitudinal, regulated assay available at scale in PPMI; p‐tau217 was not available in sufficient numbers at the concurrent AD+SAA time points required for interaction and mediation analyses.^24^, ^25^ Where applicable, α‐synuclein aggregation (SAA) results were also required at the same visit.

Not all PPMI participants had concurrent AD and SAA biomarker data because CSF assays were introduced in phases and prioritized for specific research questions. Elecsys pTau181/Aβ42 testing was initially implemented for AD‐related analyses across prodromal, sporadic and genetic cohorts, while SAA was rolled out later and targeted toward genetic enrichment studies. As a result, the biomarkers available reflects assay availability and sampling strategy rather than random selection.

Analyses were retrospective on previously collected PPMI data. We initially planned to analyze all PD (sporadic and genetic), but during dataset assembly recognized that genetic composition affects SAA variability and may modify cognitive associations. Before model fitting, we therefore set sporadic PD as the primary inference population (main‐effect/mediation) and tested AD biomarker × SAA interactions in the combined cohort with genetic‐subgroup adjustment.

### Index Visits

**Figure 1:**
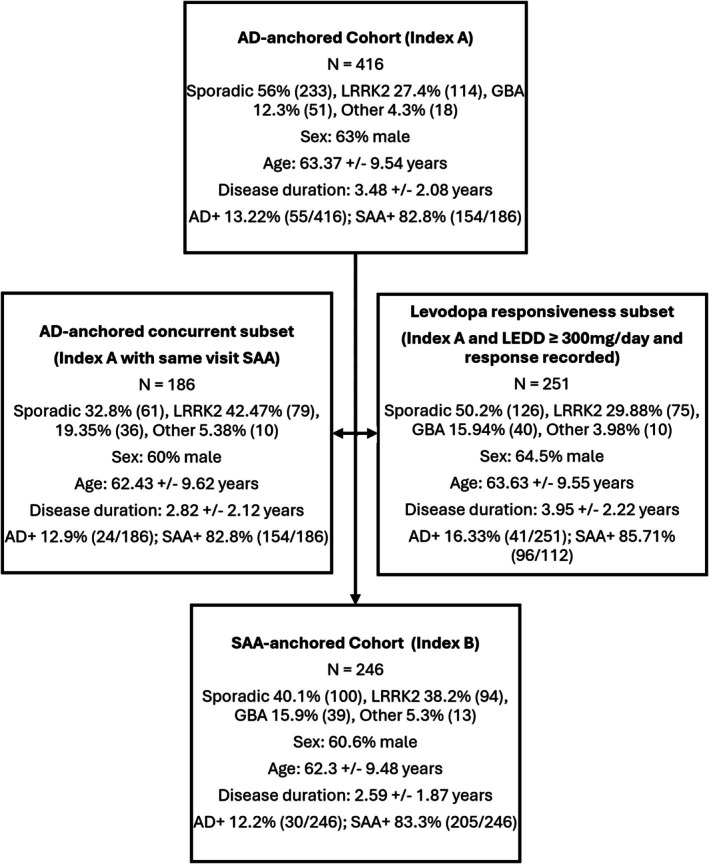
Cohort flow and indexing strategy (single cohort with nested subsets). The master cohort included 416 participants with cerebrospinal fluid (CSF) phosphorylated tau 181 to amyloid‐beta 42 ratio (pTau181/Aβ42) and clinical outcomes available at least once. Two index visits were defined for different analytic purposes. Index A (Alzheimer's disease–anchored index) was the earliest visit with Alzheimer's disease biomarkers and was used to examine the main effects of Alzheimer's disease biomarker burden, levodopa responsiveness, and mediation models. Index B (seed amplification assay–anchored index) was the earliest visit with concurrent Alzheimer's disease biomarkers and alpha‐synuclein seed amplification assay (SAA) results, and it was used for analyses testing interaction effects between Alzheimer's disease and alpha‐synuclein biomarkers. Nested subsets included an Alzheimer's disease–anchored concurrent subset (*N* = 186), which comprised participants whose Index A visit also had seed amplification assay results on the same day and was used for sensitivity analyses, and a levodopa responsiveness subset (*N* = 251), which included participants whose Index A visit also had a levodopa equivalent daily dose (LEDD) of at least 300 mg/day and Movement Disorder Society–Unified Parkinson's Disease Rating Scale (MDS‐UPDRS) Part III scores recorded in both ON and OFF medication states. Solid boxes and arrows indicate primary analyses, whereas dashed boxes and arrows indicate sensitivity analyses. In some participants, Index A and Index B coincided; in others, Index B occurred later because seed amplification assay testing was implemented after Alzheimer's disease biomarker testing. All covariates were defined at the relevant index visit. Age (mean +/− SD).

Two index visits were defined to address different analytic aims. Index A (AD‐anchored) was the earliest visit with available Elecsys pTau181/Aβ42 and cognitive outcomes (*N* = 416; sporadic 56%, LRRK2 27.4%, GBA 12.3%, other 4.3%) (Fig. 1).

Index B (SAA‐anchored) was the earliest visit where AD biomarkers and SAA were measured in the same CSF sample (N = 246; sporadic 40.1%, LRRK2 38.2%, GBA 15.9%, other 5.3%).

For clarity, neither Index A nor Index B corresponded to the PPMI enrolment visit. At both index visits, participants were typically 2–3 years into their disease course, and many were already receiving dopaminergic therapy, as reflected in the LEDD values in Table 1.

**TABLE 1:** Comparative baseline characteristics at index A and index B, stratified by sporadic and genetic Parkinson's disease

Variable	Index	All	Sporadic PD	LRRK2	GBA	Other
*N* (%)	Index A	416 (100%)	233 (56%)	114 (27.4%)	51 (12.3%)	18 (4.3%)
	Index B	246 (100%)	100 (40%)	94 (38.2%)	39 (15.9%)	13 (5.3%)
Age (years)	Index A	63.4 ± 9.5	64.2 ± 9.5	63.4 ± 8.9	61.4 ± 10.4	57.7 ± 9.9
	Index B	62.3 ± 9.48	63.1 ± 9.69	62.6 ± 8.86	61.8 ± 9.79	56.5 ± 10.2
Disease duration (years)	Index A	3.48 ± 2.08	3.29 ± 1.81	3.68 ± 2.25	3.89 ± 2.49	3.56 ± 2.68
	Index B	2.59 ± 1.87	1.85 ± 0.80	2.93 ± 2.04	3.62 ± 2.53	2.65 ± 2.11
Education (years)	Index A	15.6 ± 3.3	15.8 ± 2.6	15.1 ± 4.5	16.7 ± 3.1	14.4 ± 2.7
	Index B	15.7 ± 3.50	15.9 ± 2.54	15.2 ± 4.26	16.9 ± 3.43	14.1 ± 2.99
LEDD (mg/day)	Index A	481 ± 407	412 ± 378	557 ± 430	614 ± 413	519 ± 444
	Index B	422 ± 349	307 ± 252	482 ± 412	558 ± 315	460 ± 363
pTau181/Aβ42 ratio	Index A	0.0186 ± 0.0114	0.0187 ± 0.0112	0.0187 ± 0.0129	0.0184 ± 0.0103	0.0160 ± 0.0025
	Index B	0.0180 ± 0.0095	0.0179 ± 0.0103	0.0176 ± 0.0081	0.0198 ± 0.0119	0.0158 ± 0.0021
MoCA	Index A	26.5 ± 3.0	26.4 ± 2.9	26.3 ± 3.2	26.7 ± 2.1	26.6 ± 5.5
	Index B	26.3 ± 2.82	26.4 ± 2.50	26.1 ± 3.07	26.6 ± 1.86	26.8 ± 5.05
MDS‐UPDRS‐III	Index A	26.1 ± 11.5	27.4 ± 11.5	23.2 ± 10.8	28.0 ± 11.8	22.8 ± 10.8
	Index B	24.5 ± 11.3	26.3 ± 11.6	21.3 ± 10.5	28.9 ± 10.8	19.3 ± 9.11
GDS	Index A	2.64 ± 2.82	2.42 ± 2.62	3.08 ± 3.22	2.67 ± 2.73	2.67 ± 2.72
	Index B	2.67 ± 2.84	2.68 ± 3.13	2.79 ± 2.71	2.59 ± 2.75	2.08 ± 1.71
STAI	Index A	66.2 ± 18.5	64.7 ± 18.2	69.0 ± 18.8	66.3 ± 19.6	67.1 ± 17.7
	Index B	67.5 ± 19.5	67.0 ± 20.0	68.5 ± 19.1	66.7 ± 19.4	66.8 ± 20.1

*Note*: This table presents descriptive statistics for participants at two distinct index visits used in the study. Index A, referred to as the AD‐index, represents the earliest visit at which cerebrospinal fluid (CSF) biomarkers for Alzheimer's disease were available, specifically the ratio of phosphorylated tau 181 (pTau181) to amyloid‐beta 42 (Aβ42). Index B, referred to as the SAA‐index, represents the earliest visit at which both AD biomarkers and α‐synuclein seed amplification assay (SAA) results were measured concurrently. Participants are stratified into sporadic Parkinson's disease (PD) and genetically defined PD, including carriers of mutations in leucine‐rich repeat kinase 2 (LRRK2), glucocerebrosidase (GBA), and other genes such as α‐synuclein (SNCA) and parkin (PRKN). Variables include age, disease duration (calculated as age at visit minus age at diagnosis), years of education, and levodopa equivalent daily dose (LEDD, measured in milligrams per day). Biomarker burden is represented by the CSF pTau181/Aβ42 ratio. Cognitive function is assessed using the Montreal Cognitive Assessment (MoCA). Motor severity is measured using Part III of the Movement Disorder Society–Unified Parkinson's Disease Rating Scale (MDS‐UPDRS‐III). Mood symptoms are assessed using the Geriatric Depression Scale (GDS) and the State–Trait Anxiety Inventory (STAI). All values are presented as mean ± standard deviation, and subgroup percentages are shown in parentheses. This comparison highlights differences in demographic and clinical characteristics between the two index definitions and across genetic subgroups.

A subset of 186 participants had SAA results available at their AD‐anchored visit (Index A); this concurrent subset was used for sensitivity analyses to confirm that findings were consistent with the SAA‐anchored visit (Index B).

A separate levodopa responsiveness subset (*N* = 251) included participants at Index A with LEDD ≥300 mg/day and ON–OFF response recorded.

Sporadic PD was the primary inference population; interaction analyses were conducted in the combined cohort (all PD participants, sporadic and genetic) with adjustment for genetic subgroup.

### Biomarker Acquisition and Definitions

AD biomarker burden was defined as the CSF pTau181/Aβ42 ratio (Elecsys®), modeled continuously and secondarily as binary (AD+ if ≥0.023).^13^ α‐Synuclein aggregation was assessed by CSF SAA (Amprion protocols; 150 and 24‐h runs, as available). Only definitive Lewy body–type positive/negative results were included; MSA‐like or inconclusive patterns were excluded.

### Clinical Outcomes

Visuospatial function. The visuospatial domain was assessed with Benton's Judgment of Line Orientation (JLO). JLO‐MSSAE (MOANS age‐ and education–adjusted scaled score) was the primary visuospatial endpoint. JLO‐MSSA (MOANS age‐adjusted scaled score) and BJLOT raw total are alternate scorings of the same instrument and were analyzed as sensitivity checks. Because these three metrics are not independent endpoints, no multiplicity correction was applied within the visuospatial domain.

Secondary domains included global cognition, assessed using the Montreal Cognitive Assessment (MoCA); memory, assessed using the Hopkins Verbal Learning Test–Immediate Recall (HVLT‐IR) and Delayed Recall (HVLT‐DR); attention/working memory and processing speed, assessed using the Symbol Digit Modalities Test (SDMT) and Letter‐Number Sequencing (LNS); and semantic fluency, assessed using the Verbal Fluency Test–Animals (VLT‐ANIM) and the Semantic Fluency Test–Animals (SFTANIM). While no dedicated executive tests were available, SDMT, LNS, and fluency tasks engage executive processes. Non‐cognitive outcomes included MDS‐UPDRS Parts I–IV, GDS, STAI, and levodopa responsiveness.

Levodopa responsiveness was analyzed in a subset of participants receiving ≥300 mg/day LEDD using logistic and multinomial regression models, adjusted for demographic and clinical covariates. This threshold was selected to ensure participants were receiving a clinically meaningful dose, sufficient to elicit a reliable motor response, and aligns with dosing used in major trials such as the LEAP study.^26^, ^27^

### Statistical Analysis

Analyses examined three key questions: (1) whether AD biomarker burden was associated with cognitive outcomes, (2) whether this effect differed by α‐synuclein status, and (3) whether AD biomarkers mediated the association between age and cognition. Sensitivity analyses and power calculations are detailed in the Supplemental Methods.

### Covariates and Model Specification

Cognitive models were adjusted for age, sex, education, and MDS‐UPDRS‐III (except mediation). We did not adjust again for variables already included in the scoring derivation (age, education). Motor models included age, sex, LEDD, and disease duration (added for Parts II and IV). Mood models adjusted for age and sex. For completeness, we also repeated the mood models with additional covariates (disease duration, LEDD, and MDS‐UPDRS III/IV); these sensitivity analyses did not materially alter the results. Levodopa responsiveness models included age, sex, education, disease duration, LEDD, and MDS‐UPDRS‐III/IV. Analyses used linear regression; logistic and multinomial models were applied for levodopa responsiveness. Model assumptions were examined using representative regression models across cognitive, visuospatial, mood, and motor outcomes. Assumptions appeared reasonable and did not require transformation or alternative modeling approaches; heteroscedasticity‐robust (HC3) standard errors were used throughout.

### Multiplicity, Missing Data, and Software

Multiplicity control (BH‐FDR) was applied only across distinct cognitive domains (eg, memory, attention/working memory & processing speed, semantic fluency) and not across alternate scorings of JLO, which were prespecified as sensitivity measures. Significance after FDR correction was defined as a Benjamini–Hochberg adjusted p‐value (q‐value) < 0.05.

Analyses were complete case. Analyses used R (RStudio 2025.05.01; packages: stats, sandwich, lmtest, mediation, tidyverse).

## Results

### Cohort Flow and Baseline Characteristics

The Index A (AD‐anchored) cohort comprised 416 participants (sporadic 56%, LRRK2 27.4%, GBA 12.3%, other 4.3%). The Index B (SAA‐anchored, concurrent AD+SAA) cohort included 246 participants (sporadic 40.1%, LRRK2 38.2%, GBA 15.9%, other 5.3%). An AD‐anchored concurrent subset (Index A with same‐day SAA) contained 186 participants and supported sensitivity checks. A levodopa‐responsiveness subset (Index A with LEDD ≥ 300 mg/day and ON–OFF response recorded) included 251 participants. At Index A, 13.2% were AD+ (pTau181/Aβ42 ≥ 0.023); at Index B, 12.2% were AD+ and 83.3% were SAA+. At Index A, 96.4% of participants scored ≥21 on MoCA (n = 401), while 15 participants (3.6%) scored <21. Among those with dementia‐level MoCA scores, 14 were AD‐negative and 1 was AD‐positive. Full descriptive characteristics (age, education, LEDD, MoCA, MDS‐UPDRS, pTau181/Aβ42) are tabulated for both index definitions and by genetic subgroup (Fig. 1; Table 1 and [Link], [Link], [Link]).

Retention from Index A to Index B was 59.4% (246/416). Those retained had shorter disease duration at their Index A visit (3.13 vs 3.98 y; difference −0.85 y, 95% CI −1.24 to −0.46). Retention varied by subgroup: sporadic 42.9%, LRRK2 83.3%, GBA 76.5%, PRKN 62.5%, SNCA 100%, and LRRK2 + GBA 50%. This compositional shift explains the shorter mean disease duration at Index B (≈ 2.6 y) compared with Index A (≈ 3.48 y).

### AD Main Effects and Levodopa Responsiveness (Index A)

**Figure 2:**
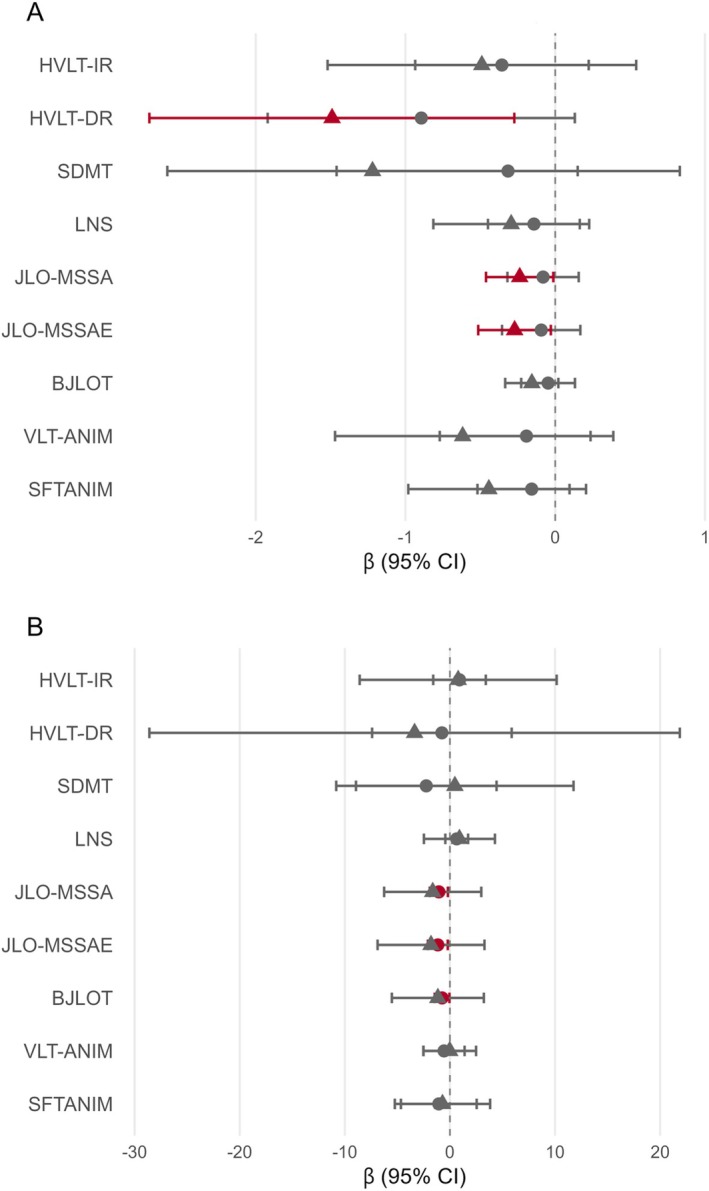
Main and interaction effects of Alzheimer's disease (AD) biomarker burden on cognition in Parkinson's disease. Panel A shows the association between continuous AD biomarker burden (cerebrospinal fluid phosphorylated tau 181 to amyloid‐β42 ratio, pTau181/Aβ42, per +0.01 unit) and cognitive outcomes at the earliest AD ascertainment visit (Index A). Panel B shows the interaction between AD biomarker burden and α‐synuclein seed amplification assay (SAA) status (positive vs negative) on the same outcomes at the earliest concurrent visit (Index B). Estimates are shown separately for the idiopathic (sporadic) cohort and the combined PD cohort. Negative β values indicate worse cognitive performance. Models were adjusted for age, sex, education, and baseline motor severity. Significant associations (*P* < 0.05) are highlighted in red. Within the visuospatial domain, JLO‐MSSAE (MOANS age and education–adjusted scaled score) was the prespecified primary endpoint. JLO‐MSSA (MOANS age‐adjusted) and BJLOT raw total are alternate scorings of the same JLO instrument and are shown as sensitivity analyses only. HVLT‐IR, Hopkins Verbal Learning Test–Immediate Recall; HVLT‐DR, Hopkins Verbal Learning Test–Delayed Recall; SDMT, Symbol Digit Modalities Test; LNS, Letter‐Number Sequencing; JLO‐MSSA, Judgment of Line Orientation–MOANS Scaled Score (age‐adjusted); JLO‐MSSAE, Judgment of Line Orientation–MOANS Scaled Score (age‐ and education‐adjusted); BJLOT, Benton Judgment of Line Orientation Test; VLT‐ANIM, Verbal Fluency Test–Animals; SFTANIM, Semantic Fluency Test–Animals.

In sporadic PD, higher CSF pTau181/Aβ42 (continuous, per +0.01) was associated with poorer delayed recall (HVLT‐DR β = −1.49, 95% CI −2.71 to −0.27; *P* = 0.017) and worse visuospatial performance. The primary visuospatial endpoint (JLO‐MSSAE) showed β = −0.271 (95% CI −0.514 to −0.0289; *P* = 0.028), and the alternate age‐adjusted scoring (JLO‐MSSA) showed β = −0.237 (95% CI −0.462 to −0.0126; *P* = 0.039. Effects on SDMT, BJLOT, MoCA, and fluency were nonsignificant (*P* ≥ 0.08–0.60). Grouping MoCA + HVLT‐DR, HVLT‐DR remained significant after BH‐FDR, whereas MoCA did not. Mood and MDS‐UPDRS I–IV domains were nonsignificant or borderline only (Fig. 2, Tables 2, S4 and 5). Binary AD positivity (≥0.023) showed no significant effects (all p ≥ 0.09), though MoCA and STAI trended lower/higher respectively.

**TABLE 2:** Main and interaction effects of Alzheimer's disease biomarker burden on cognitive outcomes in Parkinson's disease

Outcome	Sporadic PD—Main Effect β (95% CI), *P*, *N*	Combined PD—Main Effect β (95% CI), *P*, *N*	Sporadic PD—AD×SAA β (95% CI), *P*, *N*	Combined PD—AD×SAA β (95% CI), *P*, *N*
MoCA	−0.333 (−0.859, 0.192); 0.212; *N* = 233	−0.266 (−0.578, 0.046); 0.095; *N* = 416	−1.262 (−4.352, 1.828); 0.426; *N* = 100	−0.505 (−1.857, 0.847); 0.465; *N* = 246
HVLT‐IR	−0.490 (−1.520, 0.541); 0.350; *N* = 233	−0.356 (−0.935, 0.224); 0.228; *N* = 415	0.787 (−8.585, 10.158); 0.870; *N* = 100	0.912 (−1.590, 3.413); 0.476; *N* = 246
DVT‐DR	**−1.490 (−2.710, −0.273); 0.017; *N* = 233**	−0.894 (−1.920, 0.131); 0.087; *N* = 415	−3.361 (−28.593, 21.872); 0.795; *N* = 100	−0.763 (−7.404, 5.877); 0.822; *N* = 246
SDMT	−1.220 (−2.590, 0.150); 0.081; *N* = 233	−0.315 (−1.460, 0.832); 0.589; *N* = 414	0.472 (−10.820, 11.763); 0.935; *N* = 100	−2.250 (−8.938, 4.438); 0.510; *N* = 246
LNS	−0.294 (−0.814, 0.227); 0.267; *N* = 233	−0.142 (−0.449, 0.164); 0.362; *N* = 415	0.905 (−2.474, 4.285); 0.601; *N* = 100	0.648 (−0.435, 1.732); 0.242; *N* = 246
JLO‐MSSA	**−0.237 (−0.462, −0.013); 0.039; *N* = 232**	−0.081 (−0.319, 0.157); 0.504; *N* = 412	−1.635 (−6.257, 2.987); 0.490; *N* = 100	**−1.051 (−1.909, −0.194); 0.017;** *N* = **245**
JLO‐MSSAE	**−0.271 (−0.514, −0.029); 0.028; *N* = 232**	−0.093 (−0.355, 0.168); 0.484; *N* = 412	−1.796 (−6.881, 3.289); 0.491; *N* = 100	**−1.150 (−2.093, −0.207); 0.018; *N* = 245**
BJLOT	−0.156 (−0.334, 0.021); 0.084; *N* = 232	−0.047 (−0.227, 0.132); 0.603; *N* = 412	−1.146 (−5.522, 3.230); 0.609; *N* = 100	−0.745 (−1.442, −0.048); 0.037; *N* = 245
VLT‐ANIM	−0.618 (−1.470, 0.236); 0.155; *N* = 233	−0.192 (−0.771, 0.388); 0.516; *N* = 415	−0.019 (−2.530, 2.491); 0.988; *N* = 100	−0.557 (−2.511, 1.397); 0.577; *N* = 246
SFTANIM	−0.443 (−0.981, 0.096); 0.107; *N* = 233	−0.157 (−0.519, 0.206); 0.396; *N* = 415	−0.707 (−5.240, 3.825); 0.760; *N* = 100	−1.058 (−4.662, 2.546); 0.566; *N* = 246

*Note*: This table summarizes the association between Alzheimer's disease (AD) biomarker burden and cognitive performance. Main‐effect models (Index A) estimate the association between continuous AD biomarker burden—measured as the cerebrospinal fluid (CSF) phosphorylated tau to amyloid‐β42 ratio (pTau181/Aβ42, per +0.01 unit)—and cognitive outcomes at the earliest AD ascertainment visit. Interaction models (Index B) estimate the differential effect of AD biomarker burden by α‐synuclein seed amplification assay (SAA) status (positive vs negative) at the earliest concurrent visit where both biomarkers were available. Results are shown for the idiopathic Parkinson's disease (PD) subgroup (primary inference population) and for the combined PD cohort (including genetic subgroups). Cognitive domains include global cognition (Montreal Cognitive Assessment, MoCA), memory (Hopkins Verbal Learning Test–Immediate Recall, HVLT‐IR; Digit Verbal Test–Delayed Recall, DVT‐DR), attention and working memory (Symbol Digit Modalities Test, SDMT; Letter‐Number Sequencing, LNS), visuospatial function (Judgment of Line Orientation–MOANS Scaled Score, age‐adjusted [JLO‐MSSA] and age‐ and education‐adjusted [JLO‐MSSAE]; Benton Judgment of Line Orientation Test, BJLOT), and semantic fluency (Verbal Fluency Test–Animals, VLT‐ANIM; Semantic Fluency Test–Animals, SFTANIM). All models were adjusted for age, sex, years of education, and baseline motor severity (MDS‐UPDRS Part III). Interaction coefficients are expressed per 1.00 unit of pTau181/Aβ42; to compare numerically with main‐effect models, divide interaction estimates and confidence intervals by 100. Negative β values indicate worse cognitive performance. Statistically significant results (*P* < 0.05) are shown in bold.

Abbreviations: AD, Alzheimer's disease; SAA, seed amplification assay; PD, Parkinson's disease; MoCA, Montreal Cognitive Assessment; HVLT‐IR, Hopkins Verbal Learning Test–Immediate Recall; DVT‐DR, Digit Verbal Test–Delayed Recall; SDMT, Symbol Digit Modalities Test; LNS, Letter‐Number Sequencing; JLO‐MSSA, Judgment of Line Orientation–MOANS Scaled Score (age‐adjusted); JLO‐MSSAE, Judgment of Line Orientation–MOANS Scaled Score (age‐ and education‐adjusted); BJLOT, Benton Judgment of Line Orientation Test; VLT‐ANIM, Verbal Fluency Test–Animals; SFTANIM, Semantic Fluency Test–Animals.

In combined cohort PD, ADpositivity (binary, ≥ 0.023) was associated with worse delayed recall (HVLT‐DR β = −3.67, 95% CI −7.18 to −0.17; *P* = 0.040), with borderline associations for STAI and MDS‐UPDRS II–III; other cognitive domains were inconsistent.

In 251 participants receiving ≥ 300 mg/day LEDD, responsiveness was modeled as (i) continuous (% improvement in UPDRS‐III), (ii) binary (≥30% improvement), and (iii) three‐level categorical (< 20%, 20%–49%, ≥50%). Across all definitions, neither continuous AD biomarker burden (pTau181/Aβ42; β ≈ −43.0, *P* = 0.70) nor AD positivity (≥0.023; *P* ≥ 0.18) was associated with responsiveness in the total cohort or sporadic PD subgroup.

In contrast, higher MDS‐UPDRS‐III predicted less response (eg, β ≈ −0.24, *P* ≈ 0.02), while higher MDS‐UPDRS‐IV predicted greater response (β ≈ +2.6, *P* < 0.001), a pattern consistent across continuous, binary, and multinomial models. These findings indicate that levodopa responsiveness in early PD reflects motor severity and complications rather than AD co‐pathology.

### AD × SAA Interaction (Index B; Concurrent Cohort)

Primary interaction analysis focused on JLO‐MSSAE. In the combined PD cohort at the earliest concurrent visit, higher AD biomarker burden (continuous pTau181/Aβ42) interacted with SAA positivity to predict worse visuospatial performance (β = −1.15, 95% CI −2.093 to −0.207; *P* = 0.018). In sporadic PD, estimates were directionally consistent but underpowered. No other cognitive domains (MoCA; memory; attention/working memory & processing speed; semantic fluency), nor mood (GDS, STAI) or motor score (MDS‐UPDRS I–IV), showed consistent interaction effects (Fig. 2, Table 2 and S6).

### Sensitivity and Consistency Analyses

Treating JLO‐MSSA and BJLOT raw as alternate scorings of the same instrument, results were directionally similar (JLO‐MSSA β = −1.051, 95% CI −1.909 to −0.194; *P* = 0.017; BJLOT β = −0.745, 95% CI −1.443 to −0.048; *P* = 0.037). In combined‐cohort models adjusted for genetic subgroup (Sporadic/LRRK2/GBA/Other), the AD × SAA interaction at the visuospatial endpoint remained nominally significant (eg, JLO‐MSSAE *P* = 0.020), and leave‐one‐subgroup‐out analyses (dropping LRRK2 or GBA) yielded comparable estimates (JLO scaled measures *P* ≈ 0.042–0.047), indicating the signal was not driven by a single genetic subgroup despite LRRK2 contributing most SAA‐negative cases. Replication in the AD‐anchored concurrent subset (*N* = 186) showed directionally concordant effects with near‐threshold *P*‐values for JLO‐MSSA/MSSAE (*P* ≈ 0.059–0.060) and a similar, albeit non‐significant, pattern for BJLOT (*P* = 0.118).

### Effect Modification by Genetic Status

The prespecified three‐way term (AD × SAA × Genetic, sporadic vs genetic PD) was not significant for any cognitive outcome (all *P* ≥ 0.29; BH FDR q ≈ 0.95), indicating that the observed AD × SAA interaction does not differ systematically between sporadic and genetic PD.

### Power Considerations

Given the Index‐B composition, sporadic PD analyses (*n* = 100) had 80% power to detect interactions explaining ≥8% of residual variance (partial R^2^ ≥ 0.079; partial r ≥ 0.28), whereas combined cohort PD (*n* = 246) could detect ~3% (partial R^2^ ≥ 0.032; partial r ≥ 0.18). The observed visuospatial interactions in combined cohort PD accounted for ~1.8%–2.4% of residual variance, ie, below sporadic‐only detectability but within range for combined cohort analyses. To reach 80% power for effects of this magnitude in a sporadically concurrent cohort would require ~330–440 participants if SAA status was balanced; however, because sporadic PD is ≈95% SAA+, the effective information for the interaction is reduced, inflating the realistic requirement to ~950–1300. All detectable effect estimates correspond to 80% power at a two‐sided α = 0.05. These figures justify our emphasis on reporting combined cohort models with genetic adjustment.

### Mediation of Age Effects by AD Biomarker Burden (Index A; Sporadic PD)

In sporadic PD, a 10 year increase in age was associated with poorer visuospatial performance, of which ~10–14% was indirectly ‐mediated by higher pTau181/Aβ42: JLO‐MSSA (ACME = −0.068, *P* = 0.034; TE = −0.516, *P* < 0.001), JLO‐MSSAE (ACME = −0.078, *P* = 0.020; TE = −0.575, *P* < 0.001), and BJLOT (ACME = −0.046, p = 0.042; TE = −0.456, *P* < 0.001). Processing speed (SDMT) also showed modest mediation (ACME = −0.355, *P* = 0.032; TE = −4.09, *P* < 0.001). For MoCA, the total age effect was significant but the indirect effect via pTau181/Aβ42 was not significant in sporadic PD; in combined cohort PD the mediated fraction was ~13% and persisted after genetic adjustment (Table 3).

**TABLE 3:** Mediation of age‐related cognitive decline by Alzheimer's disease biomarker burden in sporadic Parkinson's disease (index A)

Outcome	*N*	ACME (95% CI)	*P*	ADE (95% CI)	*P*	TE (95% CI)	*P*	Prop. Mediated (95% CI)
JLO‐MSSA	**232**	**−0.068 (−0.132, −0.009)**	**0.034**	−0.447 (−0.748, −0.137)	0.002	−0.516 (−0.808, −0.233)	<0.001	0.13 (0.02, 0.39)
JLO‐MSSAE	**232**	**−0.078 (−0.147, −0.013)**	**0.02**	−0.497 (−0.837, −0.178)	0.002	−0.575 (−0.907, −0.269)	<0.001	0.14 (0.02, 0.38)
BJLOT	**232**	**−0.046 (−0.090, −0.003)**	**0.042**	−0.410 (−0.655, −0.214)	<0.001	−0.456 (−0.684, −0.260)	<0.001	0.10 (0.006, 0.25)
SDMT	**233**	**−0.355 (−0.761, −0.030)**	**0.032**	−3.733 (−4.865, −2.577)	<0.001	−4.089 (−5.234, −3.007)	<0.001	0.09 (0.008, 0.19)
MoCA	233	−0.094 (−0.256, 0.020)	0.122	−0.791 (−1.160, −0.470)	<0.001	−0.885 (−1.244, −0.572)	<0.001	0.11 (−0.02, 0.28)

*Note*: This table presents estimates from causal mediation analyses decomposing the total effect of age on cognitive outcomes into indirect effects operating through Alzheimer's disease (AD) biomarker burden and direct effects not explained by AD biomarkers. The mediator was the cerebrospinal fluid (CSF) phosphorylated tau to amyloid‐β42 ratio (pTau181/Aβ42), modeled as a continuous measure of AD biomarker burden. Analyses were restricted to participants with sporadic Parkinson's disease (PD) at the earliest visit with available AD biomarkers and cognitive outcomes. The treatment variable was age at visit, and the outcome variables included global cognition, visuospatial function, and processing speed. Global cognition was assessed using the Montreal Cognitive Assessment (MoCA). Visuospatial function was evaluated using the Benton Judgment of Line Orientation Test (BJLOT) and two MOANS‐scaled variants: the age‐corrected score (JLO‐MSSA) and the age‐ and education‐corrected score (JLO‐MSSAE). Processing speed was measured using the Symbol Digit Modalities Test (SDMT). Models adjusted for sex and years of education. The average causal mediation effect (ACME) represents the indirect effect of age on cognition through AD biomarker burden. The average direct effect (ADE) represents the effect of age not operating through AD biomarkers. The total effect (TE) is the sum of ACME and ADE. The proportion mediated reflects the fraction of the total effect explained by the AD biomarker pathway. All effects correspond to a 10‐year age contrast centered on the subgroup mean. Estimates were obtained using nonparametric bootstrapping with 1000 simulations. Negative values indicate worse cognitive performance with increasing age. Statistically significant ACME estimates (*P* < 0.05) are shown in bold.

### Relationship between AD and SAA Biomarkers

We investigated whether AD biomarker burden was associated with SAA positivity. Neither binary classification nor continuous modeling of the pTau181/Aβ42 ratio showed a significant association with SAA status (χ^2^ = 0.16, *P* = 0.686; logistic regression β = +0.37, *P* = 0.511 for binary; β = −0.95, *P* = 0.955 for continuous) (Table S7). These findings suggest no clear evidence of dependence between AD and SAA biomarkers in this cohort; however, the study may be underpowered to assess partially related processes. This uncertainty reinforces the rationale for modeling their interaction effects rather than assuming co‐occurrence or complete independence.

## Discussion

Our findings indicate that AD biomarker burden relates to cognitive heterogeneity in PD through both a direct association and an interaction with α‐synuclein aggregation. At the earliest AD‐anchored visit, higher CSF pTau181/Aβ42 was associated with poorer delayed recall and worse visuospatial performance in sporadic PD, even after adjusting for demographic and motor covariates. In the concurrent biomarker subset, AD burden interacted with SAA status to further impair visuospatial outcomes in combined cohort PD, with directionally similar estimates in sporadic PD but reduced precision. Finally, mediation analyses suggested that a modest but significant fraction (~10–14%) of age‐related visuospatial decline operates through AD biomarker burden. Together, these results highlight visuospatial cognition as a domain particularly sensitive to AD‐type processes in PD, both as a mediator of age effects and as a locus of synergistic pathology when Lewy‐type α‐synuclein aggregation is present. These observations align with prior PD studies linking CSF Aβ42/tau to cognitive impairment and progression, while extending them by demonstrating visuospatial sensitivity and additional impairment when SAA is positive.^7^, ^8^, ^9^, ^10^, ^11^

Our findings suggest that AD biomarker burden contributes to cognitive heterogeneity in PD through synergistic interactions with α‐synuclein aggregation, particularly in visuospatial domains. These effects were most pronounced in SAA‐positive individuals, where higher AD biomarker burden was consistently associated with poorer visuospatial performance across multiple scoring formats. Although this pattern appears domain‐specific, it may reflect the natural course of cognitive decline in early PD, where visuospatial dysfunction is often among the first affected domains. Given that the PPMI cohort predominantly includes individuals in the early stages of disease, it is plausible that synergistic effects between AD and SAA biomarkers are most detectable in visuospatial tasks at this stage. If the cohort were reevaluated at later stages or in populations with established cognitive impairment, synergistic effects might extend beyond visuospatial domains.

Our mediation analysis indicated that AD biomarker burden accounted for ~10–14% of the age–visuospatial association in PD. Although modest, this proportion is consistent with mediation magnitudes reported in related neurodegenerative contexts. For example, Ma and colleagues identified that the effect of β‐amyloid on cognition in mild cognitive impairment was mediated by downstream pathology at comparable scales, ~ 26% via tau‐related pathology and ~15–47% via other markers of neurodegeneration.^28^ In population‐based cohorts, APOE ε4 effects on late‐life cognition are partly mediated by neuroimaging markers, ~9% via white‐matter lesion volume alone and ~25% when combined with total brain tissue volume,^29^ illustrating that indirect pathways typically explain portions, not the majority, of complex clinical states. Taken together this should be viewed as informative rather than definitive, and individual level prognostic utility will require prospective, pre‐specified replication.

Although our analyses focused on visuospatial outcomes, the dual syndrome hypothesis of cognitive impairment in PD,^5^, ^6^ which distinguishes a frontostriatal executive‐attentional profile from a posterior cortical profile, provides a useful framework for interpreting our findings. This model posits that executive dysfunction represents a parallel pathway, often linked to frontostriatal dopaminergic deficits and levodopa responsiveness, whereas posterior cortical/visuospatial deficits are typically less levodopa‐responsive or show inconsistent responses across studies.^30^, ^31^, ^32^, ^33^ We could not directly assess executive dysfunction due to the absence of dedicated executive tasks in PPMI; however, exploratory patterns in processing speed (SDMT) and mediation analyses suggest that executive‐attentional processes may also be influenced by AD biomarker burden, particularly as disease progresses. The lack of strong interaction effects in these measures likely reflects both the limited sensitivity of available tools and the early disease stage of this cohort, where participants were medication‐optimized, by protocol design, potentially masking deficits in levodopa‐responsive executive pathways. Consistent with this interpretation, in our analysis levodopa responsiveness showed no association with AD biomarker burden, indicating that the AD‐visuospatial associations observed here are unlikely to reflect an acute dopaminergic response.

The AD×SAA interaction reached significance only in the combined PD cohort because this subset contained greater heterogeneity in SAA status, driven by genetic enrichment. Sporadic PD is almost uniformly SAA‐positive (~95%), leaving very few SAA‐negative cases and thus little statistical leverage to estimate an interaction. In contrast, genetic subgroups, particularly LRRK2 carriers, show substantially lower SAA positivity, creating the variation needed for the interaction term to be estimable with reasonable precision. Importantly, this pattern reflects both a power constraint and a biological reality: LRRK2 PD often exhibits reduced Lewy‐type α‐synuclein seeding,^14^, ^34^ so the concurrent presence of AD biomarker burden and SAA positivity may represent a more pathogenic state that disproportionately drives visuospatial impairment. Consistent with this, the direct association of AD biomarker burden with cognition was most evident in sporadic PD, where the main‐effect coefficient is the SAA‐positive slope; in combined group analyses, cross‐group heterogeneity may have attenuated the effect.

We anticipated concerns that the interaction might be an artifact of genetic composition. However, combined cohort models adjusted for genetic subgroup retained nominal significance for visuospatial outcomes, and leave‐one‐subgroup‐out analyses (dropping LRRK2 or GBA) produced comparable estimates (*P* ≈ 0.042–0.047). Replication in the AD‐anchored concurrent subset showed directional concordance with near‐threshold p‐values for JLO metrics. These findings indicate that the interaction was not driven by any single subgroup despite LRRK2 contributing most SAA‐negative cases. The prespecified three‐way interaction term (AD×SAA × Genetic) was non‐significant, further supporting the interpretation that the synergy reflects a generalizable biological phenomenon rather than a subgroup‐specific artifact.

We suspect the absence of significance in sporadic PD reflects under‐power rather than contradiction of the combined cohort signal. Power calculations indicated that sporadic‐only models (Index B, *n* = 100) had 80% power only for interactions explaining ≥8% of residual variance, whereas the observed visuospatial interactions accounted for ~2%. To detect effects of this magnitude in sporadic PD would require ~330–440 participants; given the near‐uniform SAA positivity, the realistic requirement inflates to ~950–1300. These figures explain why interaction testing in sporadic PD may be underpowered and why combined cohort models, while adjusted for genetic subgroup, were necessary to obtain stable estimates. Our conceptual focus was sporadic PD, but statistical inference for the interaction relied on combined‐cohort analyses due to limited SAA variability.

Motor outcomes were largely null across analyses, and levodopa responsiveness showed no association with AD biomarker burden or status under any definition (continuous, binary, or categorical). Instead, higher MDS‐UPDRS‐III predicted less response, while higher MDS‐UPDRS‐IV predicted greater response. This aligns with prior longitudinal work showing that single‐analyte tau or amyloid effects on motor progression are small even with repeated measures.^9^, ^21^ Cross‐sectionally, therefore, it is unsurprising that AD biomarkers explain little variance in motor severity or acute dopaminergic response.

Although clinical cutoffs are useful for classification, continuous modeling of the pTau181/Aβ42 ratio, a Core 1 biomarker in the NIA‐AA AT(N) framework, enhances sensitivity to subthreshold pathology and aligns with best practice in biomarker research, including plasma‐based PD studies.^12^, ^35^, ^36^ Prior work in AD and mixed cohorts shows that continuous modeling reduces misclassification and improves detection of early changes.^35^, ^37^, ^38^ These principles apply to CSF Elecsys assays, which are validated against amyloid and tau PET and recommended as quantitative measures for research and prognostic purposes.^12^, ^13^, ^37^ This approach complements binary classification, which remains standard for defining AD pathology, while recognizing that the ratio primarily reflects amyloid burden and that stronger correlations with clinical progression are seen with tau PET and other Core 2 biomarkers not available here.

Several limitations should be considered when interpreting these findings. First, the number of pTau181/Aβ42‐defined AD‐positive participants (AD+ if ≥0.023)^13^ was small (*n* = 55), limiting statistical power for detecting interaction and mediation effects when using a fixed cut‐off. Second, SAA was measured as a binary assay, restricting the ability to model interaction effects across a continuum of α‐synuclein burden. Third, our biomarker‐based models rely on a single (spot) CSF measurement obtained during a long disease course, which cannot capture temporal trajectories of AD‐type or α‐synuclein biology. CSF biomarkers offer an indirect view of brain pathology, and although validated at the group level, may not mirror changes in individuals or specific brain regions. Accordingly, biomarker positivity or negativity does not, by itself, establish or exclude histopathology in an individual. Moreover, our models do not incorporate other biological and clinical factors that likely contribute to neurodegeneration and cognitive heterogeneity in PD. While the pTau181/Aβ42 ratio is validated, it may not fully capture the complexity of AD‐related changes; additional processes such as neuroinflammation, vascular pathology, and blood–brain barrier dysfunction could also influence biomarker dynamics. The low prevalence of AD positivity likely reflects the PPMI study design, which excludes individuals with dementia at baseline and enrolls participants who are typically highly educated.^4^, ^39^ These factors, combined with the early disease stage and treatment‐optimization, may attenuate cognitive deficits and reduce the apparent prevalence of AD co‐pathology. Consequently, the observed effects may be more pronounced in less selectively recruited or more clinically heterogeneous populations. In addition, the cohort includes many LRRK2 and GBA carriers, who can exhibit heterogeneous or reduced Lewy‐type α‐synuclein pathology.^14^, ^34^ These genetic differences may alter propagation patterns and influence biomarker interactions, limiting generalisability to sporadic PD. Finally, as this is a retrospective analysis, findings may not generalize to prodromal or advanced PD stages, where biomarker interactions and domain spread could differ substantially.

Visuospatial deficits often signal early cognitive vulnerability in PD, and our results suggest that concurrent AD and α‐synuclein biomarker positivity may be associated with more pronounced visuospatial impairment. For clinicians, this supports incorporating visuospatial screening and where feasible, considering biomarker testing (when available). Biological heterogeneity may partly explain why monoclonal antibody trials targeting amyloid or tau in AD, and α‐synuclein in PD, have shown limited or variable clinical efficacy despite evidence of biomarker engagement.^40^, ^41^, ^42^, ^43^, ^44^ Accordingly, stratifying participants by biomarker‐defined subgroups, such as AD+ and/or SAA+ status, may improve the likelihood of detecting treatment effects and could be pursued as a hypothesis‐driven strategy to identify those most likely to benefit from targeted interventions.

In summary, our findings support a model in which AD and α‐synuclein biomarker burden synergistically contribute to early visuospatial impairment in PD. These results require replication, in independent datasets. If confirmed, dual biomarker testing could help identify individuals at higher risk of visuospatial decline and guide clinical trial stratification. Any prognostic use should await future prospective evidence.

## Author Roles

(1) Research Project: A. Conception. B. Organization. C. Execution; (2) Statistical Analysis: A. Design. B. Execution. C. Review and Critique; (3) Manuscript: A. Writing of the first draft. B. Review and Critique.

D.L.: 1A, 1B, 1C, 2A, 2B, 2C, 3A, 3B

M.B.: 1A, 1C, 3B

N.P.: 1A, 1B, 1C, 2A, 2C, 3A, 3B

M.L.: 1B, 3B

S.S.: 1C, 2C, 3B

R.I.: 1C, 2C, 3B

C.S.: 1C, 2C, 3B

V.F.: 1C, 3B

D.G.: 1C, 3B

## Disclosures

**Ethical Compliance Statement:** The Parkinson's Progression Markers Initiative (PPMI) 2.0 study was approved by the London—City & East Research Ethics Committee (Ethics Code: 20/LO/0900) on September 7, 2020. All participating sites obtained ethical approval. This research was conducted ethically in accordance with the World Medical Association Declaration of Helsinki. The study is registered on ClinicalTrials.gov (Identifier: NCT01141023). We confirm that we have read the Journal's position on issues involved in ethical publication and affirm that this work is consistent with those guidelines.

**Funding Sources and Conflicts of Interest:** No specific funding was received for this work. The authors declare that there are no conflicts of interests relevant to this work.

**Financial Disclosures for the Previous 12 Months:** DL reports employment with Newcastle Upon Tyne NHS Foundation Trust; consultancies with Bial; honoraria from Bial and Medtronic; royalties from Oxford University Press; and grants from Medtronic, Roche, and the Michael J. Fox Foundation. SS reports employment with Newcastle Upon Tyne NHS Foundation Trust and grants from the Michael J. Fox Foundation. CBS reports employment with Newcastle University. RI reports employment with Newcastle University and Newcastle Upon Tyne NHS Foundation Trust. VF reports employment with Newcastle Upon Tyne NHS Foundation Trust. DG reports employment with Newcastle Upon Tyne NHS Foundation Trust. ML reports employment with Newcastle Upon Tyne NHS Foundation Trust and Scottish Brain Sciences; honoraria from Roche Diagnostics and Lilly; and grants from the Academy of Medical Sciences and NIHR Newcastle Biomedical Research Centre. MB reports employment with Newcastle Upon Tyne NHS Foundation Trust and Newcastle University; and grants from NIHR, LifeArc, MDNA, MyName'5 Doddie Foundation, and UKRI EPSRC. NP reports employment with Newcastle University; advisory board participation for Hoffman‐La Roche, Abbvie, Bial, Teva Pharmaceutical, Biohaven, and Teitur Trophics; honoraria from Abbvie, GE Healthcare, and Bial; and grants from the Independent Research Fund Denmark, Danish Parkinson's Disease Association, GE Healthcare, the Michael J. Fox Foundation for Parkinson's Research, and F. Hoffman‐La Roche Inc.

## Consent to Participate

All participants provided written informed consent prior to enrolment in the study.

## Supporting information


**Data S1.** Supplementary Methods.
This section provides detailed descriptions of analytic procedures that extend beyond the main text. Topics include: (i) specification of the primary Alzheimer's disease (AD) × α‐synuclein aggregation (SAA) interaction term; (ii) pooled models with genetic subgroup adjustment; (iii) leave‐one‐subgroup‐out sensitivity analyses; (iv) effect modification by genetic status via three‐way interaction testing; (v) mediation analysis methods for decomposing age‐related cognitive effects; (vi) levodopa responsiveness modeling across continuous, binary, and categorical definitions; and (vii) power calculations for interaction detection thresholds. These details complement the summary provided in the Statistical Analysis section of the main text.

**Table S1.** (A and B) Baseline Demographics, Disease Duration, and Biomarker Status by Genetic Subgroup at Index A and Index B. These tables present baseline characteristics of participants at their earliest included visit under two distinct indexing strategies. (A) Corresponds to Index A, defined as the earliest visit at which cerebrospinal fluid (CSF) biomarkers for Alzheimer's disease were available, specifically the ratio of phosphorylated tau 181 (pTau181) to amyloid‐beta 42 (Aβ42). (B) Corresponds to Index B, defined as the earliest visit at which both AD biomarkers and α‐synuclein seed amplification assay (SAA) results were measured concurrently.
Participants are stratified into sporadic Parkinson's disease (sporadic PD) and genetically defined PD subgroups, including carriers of mutations in LRRK2 (leucine‐rich repeat kinase 2), GBA (glucocerebrosidase), SNCA (α‐synuclein), PRKN (parkin), and compound carriers with both LRRK2 and GBA mutations.
Variables include total number of participants (N), percentage of male participants, mean age in years (± standard deviation), mean disease duration in years (± standard deviation), and biomarker positivity rates. AD biomarker positivity (AD+) is defined as a CSF pTau181/Aβ42 ratio greater than 0.023, based on Roche Elecsys® assay thresholds. SAA positivity (SAA+) indicates the presence of Lewy body‐type α‐synuclein aggregation detected via Amprion's seed amplification assay. SAA positivity is calculated only among participants with available SAA data at the same visit. Disease duration is calculated as age at visit minus age at diagnosis. These tables highlight differences in demographic composition, disease stage, and biomarker prevalence across genetic subgroups and between the two index definitions. ^†^SAA positivity computed among those with SAA available in that subgroup.

**Table S2.** Baseline Characteristics, Clinical Outcomes, and Cognitive Performance at Index A, Stratified by Parkinson's Disease Subgroup. (A) Presents demographic variables including age, disease duration (calculated as age at visit minus age at diagnosis), years of education, and levodopa equivalent daily dose (LEDD) in milligrams per day. Alzheimer's disease biomarker burden is represented by the CSF ratio of phosphorylated tau 181 (pTau181) to amyloid‐beta 42 (Aβ42), measured using the Roche Elecsys® platform. (B) Reports motor and mood‐related clinical outcomes. Motor severity and complications are assessed using the Movement Disorder Society–Unified Parkinson's Disease Rating Scale (MDS‐UPDRS), including Part I (non‐motor experiences of daily living), Part II (motor experiences of daily living), Part III (motor examination), and Part IV (motor complications). Mood symptoms are evaluated using the Geriatric Depression Scale (GDS) and the State–Trait Anxiety Inventory (STAI). (C) Presents cognitive performance across multiple domains. Global cognition is assessed using the Montreal Cognitive Assessment (MoCA). Memory is measured using the Hopkins Verbal Learning Test–Immediate Recall (HVLT‐IR) and the Hopkins Verbal Learning Test–Delayed Recall (HVLT‐DR). Attention and working memory are evaluated using the Symbol Digit Modalities Test (SDMT) and the Letter‐Number Sequencing (LNS) task. Visuospatial abilities are assessed using the Judgment of Line Orientation–Motor Scaled Score Average (JLO‐MSSA), its education‐adjusted variant (JLO‐MSSAE), and the Benton Judgment of Line Orientation Test (BJLOT). Semantic fluency is measured using the Verbal Fluency Test–Animals (VLTANIM) and the Semantic Fluency Test–Animals (SFTANIM).

**Tables S3.** Baseline Characteristics, Clinical Outcomes, and Cognitive Performance at Index B, Stratified by Parkinson's Disease Subgroup. (A) Presents demographic variables including age, disease duration (calculated as age at visit minus age at diagnosis), years of education, and levodopa equivalent daily dose (LEDD) in milligrams per day. Alzheimer's disease biomarker burden is represented by the CSF ratio of phosphorylated tau 181 (pTau181) to amyloid‐beta 42 (Aβ42), measured using the Roche Elecsys® platform. (B) Reports motor and mood‐related clinical outcomes. Motor severity and complications are assessed using the Movement Disorder Society–Unified Parkinson's Disease Rating Scale (MDS‐UPDRS), including Part I (non‐motor experiences of daily living), Part II (motor experiences of daily living), Part III (motor examination), and Part IV (motor complications). Mood symptoms are evaluated using the Geriatric Depression Scale (GDS) and the State–Trait Anxiety Inventory (STAI). (C) Presents cognitive performance across multiple domains. Global cognition is assessed using the Montreal Cognitive Assessment (MoCA). Memory is measured using the Hopkins Verbal Learning Test–Immediate Recall (HVLT‐IR) and the Hopkins Verbal Learning Test–Delayed Recall (HVLT‐DR). Attention and working memory are evaluated using the Symbol Digit Modalities Test (SDMT) and the Letter‐Number Sequencing (LNS) task. Visuospatial abilities are assessed using the Judgment of Line Orientation–Motor Scaled Score Average (JLO‐MSSA), its education‐adjusted variant (JLO‐MSSAE), and the Benton Judgment of Line Orientation Test (BJLOT). Semantic fluency is measured using the Verbal Fluency Test–Animals (VLTANIM) and the Semantic Fluency Test–Animals (SFTANIM).

**Table S4.** Association Between AD Biomarker Positivity (pTau181/Aβ42 ≥ 0.023) and Clinical Outcomes at Index A. This table presents regression estimates (β) and 95% confidence intervals (CI) for the association between Alzheimer's disease (AD) biomarker positivity—defined as a cerebrospinal fluid (CSF) phosphorylated tau to amyloid‐β42 ratio (pTau181/Aβ42) of at least 0.023—and clinical outcomes at the earliest AD ascertainment visit (Index A). Results are shown for the sporadic Parkinson's disease (PD) subgroup (primary inference population) and for the full PD cohort (All PD). Global cognition was assessed using the Montreal Cognitive Assessment (MoCA). Memory was evaluated with the Hopkins Verbal Learning Test—Immediate Recall (HVLT‐IR) and the Hopkins Verbal Learning Test–Delayed Recall (HVLT‐DR). Attention and working memory were measured using the Symbol Digit Modalities Test (SDMT) and Letter‐Number Sequencing (LNS). Visuospatial function was assessed using the Benton Judgment of Line Orientation Test (BJLOT) and two MOANS‐scaled variants of the Judgment of Line Orientation: the age‐corrected score (JLO‐MSSA) and the age‐ and education‐corrected score (JLO‐MSSAE). Semantic fluency was measured using Verbal Fluency for Animals (VLT‐ANIM) and the Semantic Fluency Test—Animal Naming (SFTANIM). Mood was assessed with the Geriatric Depression Scale (GDS) and anxiety with the State–Trait Anxiety Inventory (STAI). Motor symptoms were evaluated using the Movement Disorder Society Unified Parkinson's Disease Rating Scale (MDS‐UPDRS), including Part I (non‐motor experiences of daily living), Part II (motor experiences of daily living), Part III (motor examination), and Part IV (motor complications). All cognitive models were adjusted for age, sex, years of education, and MDS‐UPDRS Part III score. Mood models were adjusted for age and sex. Motor models were adjusted for age, sex, and levodopa equivalent daily dose (LEDD), with disease duration additionally included for Parts II and IV. Negative β values indicate worse cognitive performance, while positive β values indicate greater symptom burden for mood or more severe motor impairment. Reported p‐values are unadjusted; no outcome met family‐wise false discovery rate (FDR) significance in these binary models. Statistically significant results are shown in bold.

**Table S5.** Association Between Continuous AD Biomarker Burden (pTau181/Aβ42 per + 0.01) and Clinical Outcomes at Index A. This table presents regression estimates (β) and 95% confidence intervals (CI) for the association between Alzheimer's disease (AD) biomarker burden, modeled continuously as the cerebrospinal fluid (CSF) phosphorylated tau to amyloid‐β42 ratio (pTau181/Aβ42, per 0.01 unit increase), and clinical outcomes at the earliest AD ascertainment visit (Index A). Results are shown for the sporadic Parkinson's disease (PD) subgroup (primary inference population) and for the full PD cohort (All PD). Global cognition was assessed using the Montreal Cognitive Assessment (MoCA). Memory was evaluated with the Hopkins Verbal Learning Test—Immediate Recall (HVLT‐IR) and the Hopkins Verbal Learning Test–Immediate Recall (HVLT‐DR). Attention and working memory were measured using the Symbol Digit Modalities Test (SDMT) and Letter‐Number Sequencing (LNS). Visuospatial function was assessed using the Benton Judgment of Line Orientation Test (BJLOT) and two MOANS‐scaled variants of the Judgment of Line Orientation: the age‐corrected score (JLO‐MSSA) and the age‐ and education‐corrected score (JLO‐MSSAE). Semantic fluency was measured using Verbal Fluency for Animals (VLT‐ANIM) and the Semantic Fluency Test—Animal Naming (SFTANIM). Mood was assessed with the Geriatric Depression Scale (GDS) and anxiety with the State–Trait Anxiety Inventory (STAI). Motor symptoms were evaluated using the Movement Disorder Society Unified Parkinson's Disease Rating Scale (MDS‐UPDRS), including Part I (non‐motor experiences of daily living), Part II (motor experiences of daily living), Part III (motor examination), and Part IV (motor complications). All cognitive models were adjusted for age, sex, years of education, and MDS‐UPDRS Part III score. Mood models were adjusted for age and sex. Motor models were adjusted for age, sex, and levodopa equivalent daily dose (LEDD), with disease duration additionally included for Parts II and IV. Negative β values indicate worse cognitive performance, while positive β values indicate greater symptom burden for mood or more severe motor impairment. Reported p‐values are unadjusted. Statistically significant results are shown in bold.

**Table S6.** Interaction Effects of Alzheimer's and α‐Synuclein Biomarkers on Cognitive and Motor Outcomes in Parkinson's Disease—Index B. This table presents interaction estimates (β) and 95% confidence intervals (CI) for the effect of Alzheimer's disease (AD) biomarker burden, measured continuously using the cerebrospinal fluid phosphorylated tau to amyloid‐β42 ratio, by α‐synuclein seeding amplification assay (SAA) status (positive vs. negative) on selected cognitive and motor outcomes. Results are shown for the full Parkinson's disease (PD) cohort (“All”) and the sporadic PD subgroup (“Sporadic”). All models were adjusted for age, sex, education, and baseline motor severity. Global cognition was assessed using the Montreal Cognitive Assessment (MoCA). Memory was evaluated using the Hopkins Verbal Learning Test—Immediate Recall (HVLT‐IR) and the Hopkins Verbal Learning Test–Delayed Recall (HVLT‐DR). Attention and working memory were measured using the Symbol Digit Modalities Test (SDMT) and Letter‐Number Sequencing (LNS). Visuospatial function was assessed using the Benton Judgment of Line Orientation Test (BJLOT), as well as two MOANS‐scaled variants: the age‐corrected score (JLO‐MSSA) and the age‐ and education‐corrected score (JLO‐MSSAE). Semantic fluency was measured using Verbal Fluency for Animals (VLT‐ANIM) and the Semantic Fluency Test—Animal Naming (SFTANIM). Mood and anxiety were assessed using the Geriatric Depression Scale (GDS) and the State–Trait Anxiety Inventory (STAI). Motor symptoms were evaluated using the Movement Disorder Society Unified Parkinson's Disease Rating Scale (MDS‐UPDRS) Parts I–IV.
Interaction terms reflect the differential effect of AD biomarker burden on outcome scores in SAA‐positive versus SAA‐negative individuals. Negative β values indicate worse performance or greater symptom burden in the AD+/SAA+ group. Interaction coefficients are per 1.00 unit of pTau181/Aβ42; to compare with main‐effect models (reported per 0.01), divide β and 95% CIs by 100. Statistically significant results are shown in bold.

**Table S7.** Cross‐Classification of Alzheimer's Disease and α‐Synuclein Biomarker Status in the Index B Cohort. This table presents the overlap between Alzheimer's disease (AD) biomarker status and α‐synuclein aggregation status in the cohort defined by Index B, which represents the earliest visit at which cerebrospinal fluid (CSF) biomarkers for AD and α‐synuclein seed amplification assay (SAA) results were measured concurrently. AD biomarker positivity (AD+) is defined as a CSF ratio of phosphorylated tau 181 (pTau181) to amyloid‐beta 42 (Aβ42) greater than 0.023, based on the Roche Elecsys® assay threshold. SAA positivity (SAA+) indicates the presence of Lewy body‐type α‐synuclein aggregation detected using Amprion's seed amplification assay protocol. The table shows the number and percentage of participants falling into each biomarker combination category: AD−/SAA−, AD+/SAA−, AD−/SAA+, and AD+/SAA+. These classifications form the basis for interaction analyses.
The chi‐square statistic (χ^2^ = 0.16, *P* = 0.686) was calculated using a Pearson chi‐square test applied directly to the observed 2 × 2 contingency table shown.

## Data Availability

The datasets generated and/or analyzed during the current study are freely available following written request, from https://www.ppmi-info.org/access-data-specimens/download-data.
