# Supplementary material for: Alzheimer's Biomarkers and Visuospatial Cognition in Parkinson's Disease: Modification by α‐Synuclein and Mediation of Age Effects
Source: Mov Disord Clin Pract. 2026 Mar 6;13(8):1861–75. doi: 10.1002/mdc3.70576 (PMC13339541; doi:10.1002/mdc3.70576)
Supplement: Supplementary file 1 [file MDC3-13-1861-s001.docx]

**Supplementary Table 1A and Table 1B. Baseline Demographics, Disease Duration, and Biomarker Status by Genetic Subgroup at Index A and Index B**

**Supplementary Table 1A. Index A Cohort**

| **Subgroup** | **N** | **% Male** | **Age (y) mean ± SD** | **Disease duration (y) mean ± SD** | **AD+ n/N (%)** | **SAA+ n/N† (%)** |
| --- | --- | --- | --- | --- | --- | --- |
| Sporadic | 233 | 69.1% | 64.24 ± 9.47 | 3.29 ± 1.81 | 37/233 (15.88%) | 63/65 (96.92%) |
| LRRK2 | 114 | 54.4% | 63.40 ± 8.9 | 3.68 ± 2.25 | 12/114 (10.53%) | 57/81 (70.37%) |
| GBA | 51 | 58.8% | 61.38 ± 10.36 | 3.89 ± 2.49 | 6/51 (11.76%) | 32/36 (88.89%) |
| SNCA | 6 | 33.3% | 49.50 ± 8.32 | 4.79 ± 4.07 | 0/6 (0%) | 6/6 (100%) |
| PRKN | 8 | 50% | 65.47 ± 5.78 | 2.90 ± 1.41 | 0/8 (0%) | 2/3 (66.67%) |
| LRRK2 + GBA | 4 | 25% | 54.31 ± 7.11 | 3.04 ± 1.95 | 0/4 (0%) | 1/2 (50%) |

**Supplementary Table 1B. Index B Cohort**

| **Subgroup** | **N** | **% Male** | **Age (y) mean ± SD** | **Disease duration (y) mean ± SD** | **AD+ n/N (%)** | **SAA+ n/N† (%)** |
| --- | --- | --- | --- | --- | --- | --- |
| Sporadic PD | 100 | 67% | 63.07 ± 9.69 | 1.85 ± 0.80 | 16/100 (16%) | 95/100 (95%) |
| LRRK2 | 94 | 56% | 62.63 ± 8.86 | 2.93 ± 2.04 | 8/94 (8.51%) | 63/94 (67.02%) |
| GBA | 39 | 59% | 61.78 ± 9.79 | 3.62 ± 2.53 | 6/39 (15.38%) | 36/39 (92.31%) |
| SNCA | 6 | 33% | 48.42 ± 6.98 | 3.71 ± 2.66 | 0/6 (0%) | 6/6 (100%) |
| PRKN | 5 | 60% | 64.95 ± 7.38 | 1.53 ± 0.48 | 0/5 (0%) | 4/5 (80%) |
| LRRK2 + GBA | 2 | 50% | 59.40 ± 5.39 | 2.26 ± 1.99 | 0/2 (0%) | 1/2 (50%) |

These tables present baseline characteristics of participants at their earliest included visit under two distinct indexing strategies. **Supplementary** **Table 1A** corresponds to Index A, defined as the earliest visit at which cerebrospinal fluid (CSF) biomarkers for Alzheimer’s disease were available, specifically the ratio of phosphorylated tau 181 (pTau181) to amyloid-beta 42 (Aβ42). **Supplementary** **Table 1B** corresponds to Index B, defined as the earliest visit at which both AD biomarkers and α-synuclein seed amplification assay (SAA) results were measured concurrently.

Participants are stratified into sporadic Parkinson’s disease (sporadic PD) and genetically defined PD subgroups, including carriers of mutations in LRRK2 (leucine-rich repeat kinase 2), GBA (glucocerebrosidase), SNCA (α-synuclein), PRKN (parkin), and compound carriers with both LRRK2 and GBA mutations.

Variables include total number of participants (N), percentage of male participants, mean age in years (± standard deviation), mean disease duration in years (± standard deviation), and biomarker positivity rates. AD biomarker positivity (AD+) is defined as a CSF pTau181/Aβ42 ratio greater than 0.023, based on Roche Elecsys® assay thresholds. SAA positivity (SAA+) indicates the presence of Lewy body-type α-synuclein aggregation detected via Amprion’s seed amplification assay. SAA positivity is calculated only among participants with available SAA data at the same visit. Disease duration is calculated as age at visit minus age at diagnosis.

These tables highlight differences in demographic composition, disease stage, and biomarker prevalence across genetic subgroups and between the two index definitions.

†SAA positivity computed among those with SAA available in that subgroup
