# Supplementary material for: Alzheimer's Biomarkers and Visuospatial Cognition in Parkinson's Disease: Modification by α‐Synuclein and Mediation of Age Effects
Source: Mov Disord Clin Pract. 2026 Mar 6;13(8):1861–75. doi: 10.1002/mdc3.70576 (PMC13339541; doi:10.1002/mdc3.70576)
Supplement: Supplementary file 2 [file MDC3-13-1861-s002.docx]

**Supplementary Table 7. Cross-Classification of Alzheimer’s Disease and α-Synuclein Biomarker Status in the Index B Cohort**

| **SAA Status** | **AD−** | **AD+** | **Total** |
| --- | --- | --- | --- |
| SAA− | 37 (90.2%) | 4 (9.8%) | 41 (16.7%) |
| SAA+ | 179 (87.3%) | 26 (12.7%) | 205 (83.3%) |
| Total | 216 (87.8%) | 30 (12.2%) | 246 |

This table presents the overlap between Alzheimer’s disease (AD) biomarker status and α-synuclein aggregation status in the cohort defined by Index B, which represents the earliest visit at which cerebrospinal fluid (CSF) biomarkers for AD and α-synuclein seed amplification assay (SAA) results were measured concurrently. AD biomarker positivity (AD+) is defined as a CSF ratio of phosphorylated tau 181 (pTau181) to amyloid-beta 42 (Aβ42) greater than 0.023, based on the Roche Elecsys® assay threshold. SAA positivity (SAA+) indicates the presence of Lewy body-type α-synuclein aggregation detected using Amprion’s seed amplification assay protocol. The table shows the number and percentage of participants falling into each biomarker combination category: AD−/SAA−, AD+/SAA−, AD−/SAA+, and AD+/SAA+. These classifications form the basis for interaction analyses.

The chi‑square statistic (χ² = 0.16, p = 0.686) was calculated using a Pearson chi‑square test applied directly to the observed 2×2 contingency table shown.
