# Supplementary material for: Alzheimer's Biomarkers and Visuospatial Cognition in Parkinson's Disease: Modification by α‐Synuclein and Mediation of Age Effects
Source: Mov Disord Clin Pract. 2026 Mar 6;13(8):1861–75. doi: 10.1002/mdc3.70576 (PMC13339541; doi:10.1002/mdc3.70576)
Supplement: Supplementary file 4 [file MDC3-13-1861-s004.docx]

**Supplementary Tables 3A–C. Baseline Characteristics, Clinical Outcomes, and Cognitive Performance at Index B, Stratified by Parkinson’s Disease Subgroup**

**Supplementary Table 3A: Demographics and Biomarker**

| **Category** | **Variable** | **All (n, %)** | **Sporadic PD** | **LRRK2** | **GBA** | **Other** |
| --- | --- | --- | --- | --- | --- | --- |
| Demographics | Age (years) | 246 (100%) | 100 (40%) | 94 (38.2%) | 39 (15.9%) | 13 (5.3%) |
|  | Mean ± SD | 62.3 ± 9.48 | 63.1 ± 9.69 | 62.6 ± 8.86 | 61.8 ± 9.79 | 56.5 ± 10.2 |
|  | Disease duration (years) | 246 (100%) | 100 (40%) | 94 (38.2%) | 39 (15.9%) | 13 (5.3%) |
|  | Mean ± SD | 2.59 ± 1.87 | 1.85 ± 0.80 | 2.93 ± 2.04 | 3.62 ± 2.53 | 2.65 ± 2.11 |
|  | Education (years) | 246 (100%) | 100 (40%) | 94 (38.2%) | 39 (15.9%) | 13 (5.3%) |
|  | Mean ± SD | 15.7 ± 3.50 | 15.9 ± 2.54 | 15.2 ± 4.26 | 16.9 ± 3.43 | 14.1 ± 2.99 |
|  | LEDD (mg/day) | 246 (100%) | 100 (40%) | 94 (38.2%) | 39 (15.9%) | 13 (5.3%) |
|  | Mean ± SD | 422 ± 349 | 307 ± 252 | 482 ± 412 | 558 ± 315 | 460 ± 363 |
| Biomarker | pTau181/Aβ42 ratio | 246 (100%) | 100 (40%) | 94 (38.2%) | 39 (15.9%) | 13 (5.3%) |
|  | Mean ± SD | 0.0180 ± 0.0095 | 0.0179 ± 0.0103 | 0.0176 ± 0.0081 | 0.0198 ± 0.0119 | 0.0158 ± 0.0021 |

**Supplementary Table 3B: Clinical Outcome Measures**

| **Category** | **Variable** | **All (n, %)** | **Sporadic PD** | **LRRK2** | **GBA** | **Other** |
| --- | --- | --- | --- | --- | --- | --- |
| MDS-UPDRS-III | MDS-UPDRS-I | 246 (100%) | 100 (40%) | 94 (38.2%) | 39 (15.9%) | 13 (5.3%) |
|  | Mean ± SD | 7.26 ± 4.87 | 7.10 ± 4.49 | 7.01 ± 5.23 | 8.79 ± 4.87 | 5.77 ± 4.48 |
|  | MDS-UPDRS-II | 245 (99.6%) | 99 (40.2%) | 94 (38.2%) | 39 (15.9%) | 13 (5.3%) |
|  | Mean ± SD | 7.50 ± 5.32 | 7.97 ± 5.57 | 6.57 ± 5.10 | 8.85 ± 5.16 | 6.54 ± 4.48 |
|  | MDS-UPDRS-III | 246 (100%) | 100 (40%) | 94 (38.2%) | 39 (15.9%) | 13 (5.3%) |
|  | Mean ± SD | 24.5 ± 11.3 | 26.3 ± 11.6 | 21.3 ± 10.5 | 28.9 ± 10.8 | 19.3 ± 9.11 |
|  | MDS-UPDRS-IV | 246 (100%) | 100 (40%) | 94 (38.2%) | 39 (15.9%) | 13 (5.3%) |
|  | Mean ± SD | 1.17 ± 2.26 | 0.75 ± 1.77 | 1.20 ± 2.30 | 2.26 ± 2.92 | 1.00 ± 2.27 |
| Mood | GDS | 246 (100%) | 100 (40%) | 94 (38.2%) | 39 (15.9%) | 13 (5.3%) |
|  | Mean ± SD | 2.67 ± 2.84 | 2.68 ± 3.13 | 2.79 ± 2.71 | 2.59 ± 2.75 | 2.08 ± 1.71 |
|  | STAI | 246 (100%) | 100 (40%) | 94 (38.2%) | 39 (15.9%) | 13 (5.3%) |
|  | Mean ± SD | 67.5 ± 19.5 | 67.0 ± 20.0 | 68.5 ± 19.1 | 66.7 ± 19.4 | 66.8 ± 20.1 |

**Supplementary Table 3C: Cognitive Outcome Measures**

| **Domain** | **Variable** | **All (n, %)** | **Sporadic PD** | **LRRK2** | **GBA** | **Other** |
| --- | --- | --- | --- | --- | --- | --- |
| Global Cognition | MoCA | 246 (100%) | 100 (40.7%) | 94 (38.2%) | 39 (15.9%) | 13 (5.3%) |
|  | Mean ± SD | 26.3 ± 2.82 | 26.4 ± 2.50 | 26.1 ± 3.07 | 26.6 ± 1.86 | 26.8 ± 5.05 |
| Memory | HVLT-IR | 246 (100%) | 100 (40.7%) | 94 (38.2%) | 39 (15.9%) | 13 (5.3%) |
|  | Mean ± SD | 24.3 ± 5.28 | 23.7 ± 5.37 | 24.6 ± 5.26 | 24.9 ± 4.27 | 24.0 ± 7.35 |
|  | DVT-DR | 246 (100%) | 100 (40.7%) | 94 (38.2%) | 39 (15.9%) | 13 (5.3%) |
|  | Mean ± SD | 44.6 ± 12.10 | 44.3 ± 11.53 | 44.3 ± 12.44 | 46.5 ± 11.63 | 44.2 ± 15.83 |
| Working Memory / Attention | SDMT | 246 (100%) | 100 (40.7%) | 94 (38.2%) | 39 (15.9%) | 13 (5.3%) |
|  | Mean ± SD | 40.7 ± 10.4 | 40.2 ± 9.79 | 40.6 ± 10.9 | 42.2 ± 9.44 | 40.0 ± 14.0 |
|  | LNS | 246 (100%) | 100 (40.7%) | 94 (38.2%) | 39 (15.9%) | 13 (5.3%) |
|  | Mean ± SD | 10.4 ± 2.64 | 10.7 ± 2.43 | 9.95 ± 2.82 | 11.2 ± 2.40 | 9.15 ± 2.76 |
| Visuo-spatial | JLO-MSSA | 245 (99.6%) | 100 (40.7%) | 93 (37.8%) | 39 (15.9%) | 13 (5.3%) |
|  | Mean ± SD | 12.4 ± 2.72 | 12.8 ± 2.67 | 12.1 ± 2.78 | 12.4 ± 2.61 | 11.3 ± 2.75 |
|  | JLO-MSSAE | 245 (99.6%) | 100 (40.8%) | 93 (37.8%) | 39 (15.9%) | 13 (5.3%) |
|  | Mean ± SD | 11.6 ± 2.83 | 12.0 ± 2.78 | 11.3 ± 2.87 | 11.3 ± 2.71 | 10.7 ± 3.22 |
|  | BJLOT | 245 (99.6%) | 100 (40.8%) | 93 (37.8%) | 39 (15.9%) | 13 (5.3%) |
|  | Mean ± SD | 12.5 ± 2.24 | 12.7 ± 2.26 | 12.2 ± 2.27 | 12.5 ± 2.10 | 11.7 ± 2.18 |
| Semantic Fluency | VLTANIM | 246 (100%) | 100 (40.8%) | 94 (38.2%) | 39 (15.9%) | 13 (5.3%) |
|  | Mean ± SD | 21.2 ± 5.83 | 20.9 ± 5.64 | 20.9 ± 5.62 | 23.4 ± 5.73 | 19.5 ± 7.95 |
|  | SFTANIM | 246 (100%) | 100 (40.7%) | 94 (38.2%) | 39 (15.9%) | 13 (5.3%) |
|  | Mean ± SD | 51.0 ± 11.1 | 50.4 ± 10.7 | 51.1 ± 11.3 | 53.8 ± 9.24 | 47.5 ± 16.7 |

**Supplementary Table 3A** presents demographic variables including age, disease duration (calculated as age at visit minus age at diagnosis), years of education, and levodopa equivalent daily dose (LEDD) in milligrams per day. Alzheimer’s disease biomarker burden is represented by the CSF ratio of phosphorylated tau 181 (pTau181) to amyloid-beta 42 (Aβ42), measured using the Roche Elecsys® platform.

**Supplementary Table 3B** reports motor and mood-related clinical outcomes. Motor severity and complications are assessed using the Movement Disorder Society–Unified Parkinson’s Disease Rating Scale (MDS-UPDRS), including Part I (non-motor experiences of daily living), Part II (motor experiences of daily living), Part III (motor examination), and Part IV (motor complications). Mood symptoms are evaluated using the Geriatric Depression Scale (GDS) and the State-Trait Anxiety Inventory (STAI).

**Supplementary Table 3C** presents cognitive performance across multiple domains. Global cognition is assessed using the Montreal Cognitive Assessment (MoCA). Memory is measured using the Hopkins Verbal Learning Test–Immediate Recall (HVLT-IR) and the Digit Verbal Test – Delayed Recall (DVT-DR). Attention and working memory are evaluated using the Symbol Digit Modalities Test (SDMT) and the Letter-Number Sequencing (LNS) task. Visuospatial abilities are assessed using the Judgment of Line Orientation–Motor Scaled Score Average (JLO-MSSA), its education-adjusted variant (JLO-MSSAE), and the Benton Judgment of Line Orientation Test (BJLOT). Semantic fluency is measured using the Verbal Fluency Test–Animals (VLTANIM) and the Semantic Fluency Test–Animals (SFTANIM).
