# Supplementary material for: Alzheimer's Biomarkers and Visuospatial Cognition in Parkinson's Disease: Modification by α‐Synuclein and Mediation of Age Effects
Source: Mov Disord Clin Pract. 2026 Mar 6;13(8):1861–75. doi: 10.1002/mdc3.70576 (PMC13339541; doi:10.1002/mdc3.70576)
Supplement: Supplementary file 5 [file MDC3-13-1861-s005.docx]

**Supplementary Table 6. Interaction Effects of Alzheimer’s and α-Synuclein Biomarkers on Cognitive and Motor Outcomes in Parkinson’s Disease – Index B**

| **Outcome** | **All: interaction β (95% CI)** | **All: p** | **All: N** | **Sporadic: interaction β (95% CI)** | **Sporadic: p** | **Sporadic: N** |
| --- | --- | --- | --- | --- | --- | --- |
| MDS-UPDRS I | −1.113 (−3.460, 1.234) | 0.353 | 246 | −2.142 (−16.386, 12.101) | 0.769 | 100 |
| MDS-UPDRS II | −0.271 (−4.620, 4.079) | 0.903 | 245 | −1.895 (−8.787, 4.997) | 0.591 | 99 |
| MDS-UPDRS III | −1.048 (−6.130, 4.034) | 0.686 | 246 | −1.663 (−6.651, 3.326) | 0.515 | 100 |
| MDS-UPDRS IV | −0.547 (−1.792, 0.698) | 0.39 | 246 | −0.486 (−8.487, 7.516) | 0.906 | 100 |
| GDS | −0.009 (−0.680, 0.661) | 0.978 | 246 | 0.238 (−1.762, 2.239) | 0.816 | 100 |
| STAI | 1.081 (−5.126, 7.288) | 0.733 | 246 | −4.326 (−28.978, 20.325) | 0.732 | 100 |
| MoCA | −0.505 (−1.857, 0.847) | 0.465 | 246 | −1.262 (−4.352, 1.828) | 0.426 | 100 |
| HVLT-IR | 0.912 (−1.590, 3.413) | 0.476 | 246 | 0.787 (−8.585, 10.158) | 0.87 | 100 |
| DVT-DR | −0.763 (−7.404, 5.877) | 0.822 | 246 | −3.361 (−28.593, 21.872) | 0.795 | 100 |
| SDMT | −2.250 (−8.938, 4.438) | 0.51 | 246 | 0.472 (−10.820, 11.763) | 0.935 | 100 |
| LNS | 0.648 (−0.435, 1.732) | 0.242 | 246 | 0.905 (−2.474, 4.285) | 0.601 | 100 |
| **JLO-MSSA** | **−1.051 (−1.909, −0.194)** | **0.017** | 245 | −1.635 (−6.257, 2.987) | 0.49 | 100 |
| **JLO-MSSAE** | **−1.150 (−2.093, −0.207)** | **0.018** | 245 | −1.796 (−6.881, 3.289) | 0.491 | 100 |
| **BJLOT** | **−0.745 (−1.442, −0.048)** | **0.037** | 245 | −1.146 (−5.522, 3.230) | 0.609 | 100 |
| VLT-ANIM | −0.557 (−2.511, 1.397) | 0.577 | 246 | −0.019 (−2.530, 2.491) | 0.988 | 100 |
| SFTANIM | −1.058 (−4.662, 2.546) | 0.566 | 246 | −0.707 (−5.240, 3.825) | 0.76 | 100 |

This table presents interaction estimates (β) and 95% confidence intervals (CI) for the effect of Alzheimer’s disease (AD) biomarker burden, measured continuously using the cerebrospinal fluid phosphorylated tau to amyloid-β42 ratio, by α-synuclein seeding amplification assay (SAA) status (positive vs. negative) on selected cognitive and motor outcomes. Results are shown for the full Parkinson’s disease (PD) cohort (“All”) and the sporadic PD subgroup (“Sporadic”). All models were adjusted for age, sex, education, and baseline motor severity.

Global cognition was assessed using the Montreal Cognitive Assessment (MoCA). Memory was evaluated using the Hopkins Verbal Learning Test – Immediate Recall (HVLT-IR) and the Digit Verbal Test – Delayed Recall (DVT-DR). Attention and working memory were measured using the Symbol Digit Modalities Test (SDMT) and Letter-Number Sequencing (LNS). Visuospatial function was assessed using the Benton Judgment of Line Orientation Test (BJLOT), as well as two MOANS-scaled variants: the age-corrected score (JLO-MSSA) and the age- and education-corrected score (JLO-MSSAE). Semantic fluency was measured using Verbal Fluency for Animals (VLT-ANIM) and the Semantic Fluency Test – Animal Naming (SFTANIM). Mood and anxiety were assessed using the Geriatric Depression Scale (GDS) and the State-Trait Anxiety Inventory (STAI). Motor symptoms were evaluated using the Movement Disorder Society Unified Parkinson’s Disease Rating Scale (MDS-UPDRS) Parts I–IV.

Interaction terms reflect the differential effect of AD biomarker burden on outcome scores in SAA-positive versus SAA-negative individuals. Negative β values indicate worse performance or greater symptom burden in the AD+/SAA+ group. Interaction coefficients are per 1.00 unit of pTau181/Aβ42; to compare with main‑effect models (reported per 0.01), divide β and 95% CIs by 100. **Statistically significant results are shown in bold.**
