# Supplementary material for: Alzheimer's Biomarkers and Visuospatial Cognition in Parkinson's Disease: Modification by α‐Synuclein and Mediation of Age Effects
Source: Mov Disord Clin Pract. 2026 Mar 6;13(8):1861–75. doi: 10.1002/mdc3.70576 (PMC13339541; doi:10.1002/mdc3.70576)
Supplement: Supplementary file 7 [file MDC3-13-1861-s007.docx]

# Supplemental Methods

## Primary Interaction Term

The primary exposure was Alzheimer’s disease (AD) biomarker burden, measured as the CSF pTau181/Aβ42 ratio (Elecsys® platform), modeled continuously. α-Synuclein aggregation status (SAA) was coded as a binary variable (positive/negative) based on Amprion seed amplification assay results. The main parameter of interest was the AD × SAA interaction term, which tests whether the association between AD biomarker burden and cognitive performance differs by SAA status.

## Pooled Model with Genetic Subgroup Adjustment

To assess whether the AD × SAA interaction was robust to genetic composition, we fitted pooled linear regression models across all PD participants in the concurrent subset (Index B), adjusting for genetic subgroup (Idiopathic, LRRK2, GBA, Other) as an additional covariate. These models used the same covariate structure as the primary idiopathic-only analyses. We report the interaction coefficient (β), standard error, 95% confidence interval, and p-value for each cognitive outcome. Multiplicity was addressed by applying Benjamini–Hochberg false discovery rate (BH-FDR) correction within the cognitive outcome family.

## Leave-One-Subgroup-Out Sensitivity Analyses

To evaluate whether the pooled interaction effect was driven by a single genetic subgroup, we repeated the pooled models after sequentially excluding LRRK2 carriers and GBA carriers. This leave-one-subgroup-out approach provides a stability check for the AD × SAA interaction under varying cohort compositions.

## Effect Modification by Genetic Status

We tested a pre-specified three-way interaction (AD biomarker burden × SAA × Genetic) to formally assess whether genetic status modifies the AD × SAA effect. Genetic status was coded as a binary indicator (Idiopathic vs Genetic PD). For each cognitive outcome, we estimated the three-way interaction term and its p-value, applying BH-FDR correction within the cognitive family. In addition, we derived stratum-specific simple slopes for the AD × SAA interaction within Idiopathic and Genetic PD strata, with 95% confidence intervals, to aid interpretation. These were reported descriptively, as the formal three-way test provides the primary inference on effect modification.

## Mediation Analysis Details

Mediation models estimated natural direct and indirect effects of age on cognition via AD biomarker burden using nonparametric bootstrap (1,000 simulations). UPDRS-III was excluded from mediator and outcome models to avoid post-treatment bias. Assumptions include no unmeasured confounding of age→mediator or mediator→outcome, and no mediator–outcome confounders affected by age. Age is not manipulable; results should be interpreted as decomposition of age-related associations rather than causal effects. Proportion mediated is most interpretable when the total effect is significant and not near zero; for outcomes with borderline total effects (e.g., DVT-DR), mediation estimates should be considered exploratory.

## Levodopa Responsiveness Analyses

Levodopa responsiveness was assessed in participants receiving ≥300 mg/day of LEDD. Responsiveness was modeled continuously (percentage improvement), as a binary outcome (≥30% improvement), and using a three-level categorical grouping (<20%, 20–49%, ≥50%). Logistic and multinomial logistic regression models were adjusted for age, sex, education, disease duration, LEDD, and MDS-UPDRS Parts III (OFF) and IV. Exploratory stratification by genetic status was performed.

## Power Calculations

We estimated detectable effect sizes for AD × SAA interaction models using observed sample sizes and degrees of freedom. In idiopathic PD (n ≈ 100), models had 80% power at α = 0.05 to detect an interaction explaining ≥8% of residual variance (partial R² ≥ 0.079; partial r ≥ 0.28). In pooled PD (n ≈ 246), the detectable threshold was ~3% (partial R² ≥ 0.032; partial r ≥ 0.18). Observed effects accounted for ~1.8–2.4% of residual variance, which is biologically meaningful but below the idiopathic-only detection threshold.
