# Supplementary material for: Alzheimer's Biomarkers and Visuospatial Cognition in Parkinson's Disease: Modification by α‐Synuclein and Mediation of Age Effects
Source: Mov Disord Clin Pract. 2026 Mar 6:10.1002/mdc3.70576. Online ahead of print. doi: 10.1002/mdc3.70576 (PMC13339541; doi:10.1002/mdc3.70576)
Supplement: Supplementary file 6 — Table S5. Association Between Continuous AD Biomarker Burden (pTau181/Aβ42 per + 0.01) and Clinical Outcomes at Index A. This table presents regression estimates (β) and 95% confidence intervals (CI) for the association between Alzheimer's disease (AD) biomarker burden, modeled continuously as the cerebrospinal fluid (CSF) phosphorylated tau to amyloid‐β42 ratio (pTau181/Aβ42, per 0.01 unit increase), and clinical outcomes at the earliest AD ascertainment visit (Index A). Results are shown for the sporadic Parkinson's disease (PD) subgroup (primary inference population) and for the full PD cohort (All PD). Global cognition was assessed using the Montreal Cognitive Assessment (MoCA). Memory was evaluated with the Hopkins Verbal Learning Test—Immediate Recall (HVLT‐IR) and the Hopkins Verbal Learning Test–Immediate Recall (HVLT‐DR). Attention and working memory were measured using the Symbol Digit Modalities Test (SDMT) and Letter‐Number Sequencing (LNS). Visuospatial function was assessed using the Benton Judgment of Line Orientation Test (BJLOT) and two MOANS‐scaled variants of the Judgment of Line Orientation: the age‐corrected score (JLO‐MSSA) and the age‐ and education‐corrected score (JLO‐MSSAE). Semantic fluency was measured using Verbal Fluency for Animals (VLT‐ANIM) and the Semantic Fluency Test—Animal Naming (SFTANIM). Mood was assessed with the Geriatric Depression Scale (GDS) and anxiety with the State–Trait Anxiety Inventory (STAI). Motor symptoms were evaluated using the Movement Disorder Society Unified Parkinson's Disease Rating Scale (MDS‐UPDRS), including Part I (non‐motor experiences of daily living), Part II (motor experiences of daily living), Part III (motor examination), and Part IV (motor complications). All cognitive models were adjusted for age, sex, years of education, and MDS‐UPDRS Part III score. Mood models were adjusted for age and sex. Motor models were adjusted for age, sex, and levodopa equivalent daily dose (LEDD), with disease durat [file MDC3-9999-0-s001.docx]

**Supplementary Table 5. Phase 1 – Association Between Continuous AD Biomarker Burden (pTau181/Aβ42 per +0.01) and Clinical Outcomes at Index A**

| **Domain** | **Outcome** | **Sporadic β (95% CI)** | **p (Idio)** | **N (Idio)** | **All PD β (95% CI)** | **p (All)** | **N (All)** |
| --- | --- | --- | --- | --- | --- | --- | --- |
| MDS-UPDRS Score | Part I | 0.664 (-0.0271, 1.35) | 0.06 | 233 | 0.372 (-0.182, 0.927) | 0.188 | 416 |
|  | Part II | 0.773 (-0.0368, 1.58) | 0.061 | 232 | 0.406 (-0.214, 1.03) | 0.199 | 415 |
|  | Part III | 0.838 (-0.853, 2.53) | 0.33 | 233 | 0.702 (-0.649, 2.05) | 0.308 | 416 |
|  | Part IV | -0.0439 (-0.268, 0.18) | 0.7 | 233 | -0.102 (-0.255, 0.0514) | 0.192 | 416 |
| Mood | GDS | 0.265 (-0.0428, 0.573) | 0.091 | 233 | 0.178 (-0.113, 0.47) | 0.23 | 416 |
|  | STAI | 1.44 (-1.3, 4.18) | 0.301 | 233 | 0.678 (-1.07, 2.42) | 0.445 | 416 |
| Global Cognition | MoCA | -0.333 (-0.859, 0.192) | 0.212 | 233 | -0.266 (-0.578, 0.0463) | 0.095 | 416 |
| Memory | HVLT-IR | -0.49 (-1.52, 0.541) | 0.35 | 233 | -0.356 (-0.935, 0.224) | 0.228 | 415 |
|  | **DVT-DR** | **-1.49 (-2.71, -0.273)** | **0.017** | **233** | -0.894 (-1.92, 0.131) | 0.087 | 415 |
| Working memory/ Attention | SDMT | -1.22 (-2.59, 0.15) | 0.081 | 233 | -0.315 (-1.46, 0.832) | 0.589 | 414 |
|  | LNS | -0.294 (-0.814, 0.227) | 0.267 | 233 | -0.142 (-0.449, 0.164) | 0.362 | 415 |
| Visuo-spatial | **JLO-MSSA** | **-0.237 (-0.462, -0.0126)** | **0.039** | **232** | -0.081 (-0.319, 0.157) | 0.504 | 412 |
|  | **JLO-MSSAE** | **-0.271 (-0.514, -0.0289)** | **0.028** | **232** | -0.0932 (-0.355, 0.168) | 0.484 | 412 |
|  | BJLOT | -0.156 (-0.334, 0.0211) | 0.084 | 232 | -0.0474 (-0.227, 0.132) | 0.603 | 412 |
| Semantic Fluency | VLT-ANIM | -0.618 (-1.47, 0.236) | 0.155 | 233 | -0.192 (-0.771, 0.388) | 0.516 | 415 |
|  | SFTANIM | -0.443 (-0.981, 0.0956) | 0.107 | 233 | -0.157 (-0.519, 0.206) | 0.396 | 415 |

This table presents regression estimates (β) and 95% confidence intervals (CI) for the association between Alzheimer’s disease (AD) biomarker burden, modeled continuously as the cerebrospinal fluid (CSF) phosphorylated tau to amyloid‑β42 ratio (pTau181/Aβ42, per 0.01 unit increase), and clinical outcomes at the earliest AD ascertainment visit (Index A). Results are shown for the sporadic Parkinson’s disease (PD) subgroup (primary inference population) and for the full PD cohort (All PD).

Global cognition was assessed using the Montreal Cognitive Assessment (MoCA). Memory was evaluated with the Hopkins Verbal Learning Test – Immediate Recall (HVLT‑IR) and the Digit Verbal Test – Delayed Recall (DVT‑DR). Attention and working memory were measured using the Symbol Digit Modalities Test (SDMT) and Letter‑Number Sequencing (LNS). Visuospatial function was assessed using the Benton Judgment of Line Orientation Test (BJLOT) and two MOANS‑scaled variants of the Judgment of Line Orientation: the age‑corrected score (JLO‑MSSA) and the age‑ and education‑corrected score (JLO‑MSSAE). Semantic fluency was measured using Verbal Fluency for Animals (VLT‑ANIM) and the Semantic Fluency Test – Animal Naming (SFTANIM).

Mood was assessed with the Geriatric Depression Scale (GDS) and anxiety with the State‑Trait Anxiety Inventory (STAI). Motor symptoms were evaluated using the Movement Disorder Society Unified Parkinson’s Disease Rating Scale (MDS‑UPDRS), including Part I (non‑motor experiences of daily living), Part II (motor experiences of daily living), Part III (motor examination), and Part IV (motor complications).

All cognitive models were adjusted for age, sex, years of education, and MDS‑UPDRS Part III score. Mood models were adjusted for age and sex. Motor models were adjusted for age, sex, and levodopa equivalent daily dose (LEDD), with disease duration additionally included for Parts II and IV. Negative β values indicate worse cognitive performance, while positive β values indicate greater symptom burden for mood or more severe motor impairment. Reported p‑values are unadjusted. **Statistically significant results are shown in bold.**
