# Supplementary material for: Alzheimer's Biomarkers and Visuospatial Cognition in Parkinson's Disease: Modification by α‐Synuclein and Mediation of Age Effects
Source: Mov Disord Clin Pract. 2026 Mar 6:10.1002/mdc3.70576. Online ahead of print. doi: 10.1002/mdc3.70576 (PMC13339541; doi:10.1002/mdc3.70576)
Supplement: Supplementary file 8 — Table S7. Cross‐Classification of Alzheimer's Disease and α‐Synuclein Biomarker Status in the Index B Cohort. This table presents the overlap between Alzheimer's disease (AD) biomarker status and α‐synuclein aggregation status in the cohort defined by Index B, which represents the earliest visit at which cerebrospinal fluid (CSF) biomarkers for AD and α‐synuclein seed amplification assay (SAA) results were measured concurrently. AD biomarker positivity (AD+) is defined as a CSF ratio of phosphorylated tau 181 (pTau181) to amyloid‐beta 42 (Aβ42) greater than 0.023, based on the Roche Elecsys® assay threshold. SAA positivity (SAA+) indicates the presence of Lewy body‐type α‐synuclein aggregation detected using Amprion's seed amplification assay protocol. The table shows the number and percentage of participants falling into each biomarker combination category: AD−/SAA−, AD+/SAA−, AD−/SAA+, and AD+/SAA+. These classifications form the basis for interaction analyses. The chi‐square statistic (χ2 = 0.16, P = 0.686) was calculated using a Pearson chi‐square test applied directly to the observed 2 × 2 contingency table shown. [file MDC3-9999-0-s005.docx]

**Supplementary Table 7. Cross-Classification of Alzheimer’s Disease and α-Synuclein Biomarker Status in the Index B Cohort**

| **SAA Status** | **AD−** | **AD+** | **Total** |
| --- | --- | --- | --- |
| SAA− | 37 (90.2%) | 4 (9.8%) | 41 (16.7%) |
| SAA+ | 179 (87.3%) | 26 (12.7%) | 205 (83.3%) |
| Total | 216 (87.8%) | 30 (12.2%) | 246 |

This table presents the overlap between Alzheimer’s disease (AD) biomarker status and α-synuclein aggregation status in the cohort defined by Index B, which represents the earliest visit at which cerebrospinal fluid (CSF) biomarkers for AD and α-synuclein seed amplification assay (SAA) results were measured concurrently. AD biomarker positivity (AD+) is defined as a CSF ratio of phosphorylated tau 181 (pTau181) to amyloid-beta 42 (Aβ42) greater than 0.023, based on the Roche Elecsys® assay threshold. SAA positivity (SAA+) indicates the presence of Lewy body-type α-synuclein aggregation detected using Amprion’s seed amplification assay protocol. The table shows the number and percentage of participants falling into each biomarker combination category: AD−/SAA−, AD+/SAA−, AD−/SAA+, and AD+/SAA+. These classifications form the basis for interaction analyses.

The chi‑square statistic (χ² = 0.16, p = 0.686) was calculated using a Pearson chi‑square test applied directly to the observed 2×2 contingency table shown.
